# MicroRNA-513b-5p targets COL1A1 and COL1A2 associated with the formation and rupture of intracranial aneurysm

**DOI:** 10.1038/s41598-021-94116-5

**Published:** 2021-07-21

**Authors:** Zheng Zheng, Yan Chen, Yinzhou Wang, Yongkun Li, Qiong Cheng

**Affiliations:** 1 Shengli Clinical Medical College, Fujian Medical University, Fuzhou, 350001 People’s Republic of China; 2 The Department of Neurology, Fujian Provincial Hospital, Fuzhou, No. 134, Dongjie road, Fuzhou, 350001 People’s Republic of China; 3 The Department of Geriatric Medicine, Fujian Provincial Hospital, Fuzhou, 350001 People’s Republic of China

**Keywords:** Cell biology, Genetics, Molecular biology, Biomarkers

## Abstract

Collagen-type I alpha 1 chain (COL1A1) and COL1A2 are abnormally expressed in intracranial aneurysm (IA), but their mechanism of action remains unclear. This study was performed to investigate the mechanism of COL1A1 and COL1A2 affecting the occurrence and rupture of IA. Quantitative real-time polymerase chain reaction was used to measure the expression of hsa-miR-513b-5p, COL1A1, COL1A2, TNF-α, IL-6, MMP2, MMP3, MMP9 and TIMP4 in patients with ruptured IA (RA) (n = 100), patients with un-ruptured IA (UA) (n = 100), and controls (n = 100). Then, human vascular smooth muscle cells (HASMCs) were cultured, and dual luciferase reporter assay was performed to analyse the targeting relationship between miR-513b-5p and COL1A1 or COL1A2. The effects of the miR-513b-5p mimic and inhibitor on the proliferation, apoptosis, and death of HASMC and the RIP1-RIP3-MLKL and matrix metalloproteinase pathways were also explored. The effect of silencing and over-expression of COL1A1 and COL1A2 on the role of miR-513b-5p were also evaluated. Finally, the effects of TNF-α on miR-513b-5p targeting COL1A1 and COL1A2 were tested. Compared with those in the control group, the serum mRNA levels of miR-513b-5p, IL-6 and TIMP4 were significantly decreased in the RA and UA groups, but COL1A1, COL1A2, TNF-α, IL-1β, MMP2, MMP3 and MMP9 were significantly increased (*p* < 0.05). Compared with those in the UA group, the expression of COL1A1, COL1A2, TNF-α, IL-1β and MMP9 was significantly up-regulated in the RA group (*p* < 0.05). Results from the luciferase reporter assay showed that COL1A1 and COL1A were the direct targets of miR-513b-5p. Further studies demonstrated that miR-513b-5p targeted COL1A1/2 to regulate the RIP1-RIP3-MLKL and MMP pathways, thereby enhancing cell death and apoptosis. Over-expression of COL1A1 or COL1A2, rather than silencing COL1A1/2, could improve the inhibitory effect of miR-513b-5p on cell activity by regulating the RIP1-RIP3-MLKL and MMP pathways. Furthermore, over-expression of miR-513b-5p and/or silencing COL1A1/2 inhibited the TNF-α-induced cell proliferation and enhanced the TNF-α-induced cell death and apoptosis. The mechanism may be related to the inhibition of collagen I and TIMP4 expression and promotion of the expression of RIP1, p-RIP1, p-RIP3, p-MLKL, MMP2 and MMP9. MiR-513b-5p targeted the inhibition of COL1A1/2 expression and affected HASMC viability and extracellular mechanism remodelling by regulating the RIP1-RIP3-MLKL and MMP pathways. This process might be involved in the formation and rupture of IA.

## Introduction

Intracranial aneurysm (IA) is a common cerebrovascular disease, and its incidence has been increasing year by year^1,2^. Spontaneous subarachnoid haemorrhage (SAH) caused by IA and cerebral haemorrhage induced by a rupture have high mortality rates^3–6^. Although nerve intervention and microscopy techniques have been developed, the disability and fatality rate of IA remain as high as 30–40%^7^. Previous studies have shown that haemodynamics, aneurysm morphology, vascular wall degeneration, immune system activation and smoking may play roles in the development and rupture of IA^2,5,8^. However, huge challenges still exist in the prevention and treatment of IA.

Recent studies have shown that microRNA (miRNA) plays a regulatory role in the formation and rupture of IA^9,10^. The miRNA is a small non-coding RNA, which is incorporated into the RNA-induced silencing complex and preferentially binds to the 3ʹ untranslated region (3ʹ-UTR) of the target mRNA to inhibit the gene expression and protein translation^11–13^. In the past decade, genetic research on IA has attracted great interest, especially collagen-related genes^14–17^. Single nucleotide polymorphisms of COL1A2 and COL3A1 are risk factors for IA^18–20^. Studies have shown that COL1A1 and COL1A2 are involved in regulating the synthesis of collagen I^21^, and COL1A1 and COL3A1 can regulate the cell activity and inflammation^22^. Inflammation, extracellular matrix reconstruction, and depletion of smooth muscle cells play key roles in the formation and rupture of aneurysm^23^. For example, the vascular smooth muscle cell metastasise to induce vascular inflammation, which promote the occurrence and development of IA^2^. In this process, matrix metalloproteinases (MMPs) regulate vascular inflammation^24^, receptor interacting serine/threonine kinase 1 (RIP1)/RIP3 induces necroptosis and inflammation of the smooth muscle cell, which are involved in the pathogenesis of aneurysms^23,25^.

Cell activity and MMPs can be regulated by miR-513b-5p in ovarian cancer^26^. The results from Targetscan and Starbase showed that miR-513b-5p has binding sites with COL1A1-3ʹUTR and COL1A2-3ʹUTR. Hence, we hypothesise that miR-513b-5p targeting of COL1A1 and COL1A2 may regulate IA by mediating the death and inflammation of smooth muscle cells. The results further confirmed the role of miR-513b-5p regulation of the RIP1/RIP3/MLKL and MMP pathways in IA.

## Materials and methods

### Clinical samples

The study population included patients admitted to the Neurology Department at the Fujian Provincial Hospital from Jan 2018 to Jan 2020. A total of 100 cases of un-ruptured IA, 100 cases of ruptured IA and 100 controls, who were age/gender-matched patients without IA symptoms or history, were included in this study. All participants were examined by computed tomography (Somatom Definition, SIEMENS, Germany) and digital subtraction angiography (DSA) (Artis Zee Leiling, SIEMENS, Germany). The locations and size of the IA were determined by DSA. All control participants had negative angiography results. Blood samples were collected once within 2 days after the rupture of the aneurysm. To exclude possible interference caused by DSA and treatment, all samples were taken before DSA and treatments. The patients were checked whether they had fasted for more than 10 h before blood collection. Fasting-blood samples (10 mL) were collected from all patients and put into a gold-top serum-separating tube (Fukang Medical Product Company, Cangzhou, China). The blood samples were processed to extract the serum within 2 h and then stored at − 80 °C until analysis. The study was approved by the Ethics Committee of the Fujian Provincial Hospital (Fuzhou, China) and carried out in accordance with the Declaration of Helsinki. All participants or guardians signed informed consent forms before the samples were collected.

Inclusion criteria of patients with IA were as follows: (1) age range of 20–80 years; (2) suitable for cerebrovascular intervention operation; (3) IA identified by DAS; and (4) the patients or guardians understood this research and signed the informed consent.

Exclusion criteria were as follows: (1) refusal to sign informed consent form; (2) severe hypertension, systolic blood pressure ≥ 200 mmHg and diastolic blood pressure ≥ 110 mmHg; (3) chronic kidney disease of stage ≥ 3; (4) with liver function impairment, such as chronic hepatitis and cirrhosis; (5) platelet count of < 1 × 10^11^/L or coagulation dysfunction; (6) myocardial infarction, heart failure and other severe cardiopathy; (7) muscular dystrophy, myasthenia gravis, myositis and other muscle and neuromuscular diseases; (8) hyperthyroidism and uncontrolled diabetes; (9) systemic infections, such as pneumonia and urinary tract infections; (10) tumour history; and (11) failure to provide clinical data.

Inclusion criteria for the control group were as follows: (1) age range of 20–80 years; (2) no family history of IA and no history of intracranial haemorrhage, such as SAH; (3) DSA examination to exclude IA and SAH; (4) no relationship with each other; and (5) signed the informed consent.

### Isolation of total RNA and qPCR analysis

Before RNA extraction, each sample was added with 100 ng of exogenous and synthetic *Caenorhabditis elegans* miR-39. The total RNA was extracted from the serum samples by using a mirVana PARIS Kit (Invitrogen, USA). According to the manufacturer’s protocol, the total RNA from the cells was isolated using an Ezna Total RNA Kit II (Omega Bio-tek, USA). The miRNA was purified using miRNeasy Mini Kit (Qiagen, Germany) and was specifically reverse transcription using TaqMan MicroRNA Reverse Transcription Kit (Applied Biosystems, USA). The mRNA was reverse transcribed using the Reverse Transcription System Kit (Takara, Japan). The reaction conditions were 25 °C for 5 min, 42 °C for 60 min and 70 °C for 15 min. The resultant complementary DNA was used as a template for the PCR experiment.

Then, 2.0 μL of the reverse transcription product, 12.5 μL of SYBR Green qPCR Master Mix (Promega, USA), 2.0 μL of the forward and reverse primers, and 8.5 μL of nuclease-free water (Solarbio, China) were used for real-time PCR reaction. The samples were put into the ABI 7500 PCR system (ABI, USA) to detect the RNA expression and calculated by the 2^−△△*Ct*^ method. The PCR reaction included 40 cycles of PCR amplification, pre-degeneration at 95 °C for 5 min and final extension at 95 °C for 15 s. Each cycle consisted of denaturation at 95 °C for 10 s, annealing at 60 °C for 30 s and extension at 72 °C for 30 s. The level of miRNA expression was normalised to cel-miR-39 (Sangon Biotech, China), and other mRNA expression level was normalised to GAPDH. The primers were designed by the Primer Premier 5.0 software (Premier, Canada) and synthesised by Sangon Biotech (Shanghai, China) (Table 1).

**Table 1:** Primers information.

Gene name	Sequence
**COL1A1**	
FORWARD	TTTGGATGGTGCCAAGGGAG
REVERSE	CACCATCATTTCCACGAGCA
**COL1A2**	
FORWARD	ACTGTAAGAAAGGGCCCAGC
**REVERSE**	AGCAAAGTTCCCACCGAGAC
miR-513b-5p	
FORWARD	CGCGTTCACAAGGAGGTGTC
REVERSE	AGTGCAGGGTCCGAGGTATT
RT Primer	GTCGTATCCAGTGCAGGGTCCGAGGTATTCGCACTGGATACGACATAAAT
**TNF-α**	
FORWARD	CATCCAACCTTCCCAAACGC
REVERSE	CGAAGTGGTGGTCTTGTTGC
**IL-1β**	
FORWARD	GCAGAAGTACCTGAGCTCGC
REVERSE	CTTGCTGTAGTGGTGGTCGG
**IL-6**	
FORWARD	AATGAGGAGACTTGCCTGGTG
REVERSE	GAGGGAATGAGGACACACCC
**MMP2**	
FORWARD	GCTGCATCCAGACTTCCTCA
REVERSE	AGGTCCTGGCAATCCCTTTG
**MMP3**	
FORWARD	CACTCACAGACCTGACTCGG
REVERSE	GAGTCAGGGGGAGGTCCATA
**MMP9**	
FORWARD	GATCATTCCTCAGTGCCGGA
REVERSE	GACCATAGAGGTGCCGGATG
**TIMP4**	
FORWARD	CTGCCTCCCAAACCCCATTA
REVERSE	CGCCATTTCTCCCCTACCAG
**GAPDH**	
FORWARD	GTCATCCCTGAGCTGAACGG
REVERSE	CCACCTGGTGCTCAGTGTAG

COL1A1, Collagen type I alpha 1 chain; TNF-α, Tumor necrosis factor alpha; IL-1β, Interleukin (IL) 1 beta; MMP2, Matrix metalloproteinases (MMP) 2; TIMP4, Tissue inhibitor of metallopeptidase 4.

### Cell culture

The human vascular smooth muscle cell (HASMC) line was purchased from the Shanghai Institute of Biochemistry and Cell Biology of the Chinese Academy of Science (Shanghai, China). The cells were cultured in DMEM (Gibco, Grand Island, NY) containing 10% foetal bovine serum (PAN biotech, Germany) and maintained at 37 °C in a humidified 5% CO_2_ incubator (SANYO, Osaka, Japan). The fresh growth medium was changed routinely every day, until the cells became confluent.

### Cell transfection

The HASMCs were seeded in 24-well plates with a density of 1 × 10^5^ cells/well. Lipofectamine 2000 Transfection Reagent (Invitrogen, USA) was used for cell transfection. Hsa-miR-513b-5p mimic and inhibitor (Zolgene Biotechnology Co., Ltd, Fuzhou, China) were used to achieve differential miRNA expression. When the cells grew to 30–50% confluence, hsa-miR-513b-5p mimic (20 nM), hsa-miR-513b-5p inhibitor (40 nM), and negative control were used to transfect the HASMCs. After 48 h of transfection, the cells were collected and examined.

HASMCs were seeded in 6-well plates with a density of 5 × 10^5^ cells/well. The plasmids of siRNA-COL1A1, siRNA COL1A2 and over-expression plasmid of OE-COL1A1 and OE-COL1A2 were purchased from Zolgene Biotechnology Co., Ltd. (Fuzhou, China). The sequences were as follows: negative control of COL1A1 siRNA was 5ʹ-GCTCACGCTTTCTCACTTT-3ʹ, siRNA-COL1A1 was 5ʹ-GCTCAGCACTTTCCTCTTT-3ʹ, negative control of COL1A2 siRNA was 5ʹ-GCAATTACCTCGATCTATT-3ʹ, and siRNA-COL1A1 was 5ʹ-GCACTTTACCAGCTTAATT-3ʹ. When the cells grew to 60–70% confluence, Lipofectamine 2000 Transfection Reagent was used for transfecting the HASMCs. The final concentration of the siRNA and OE-RNA was 50 nM. After transfection for 48 h, the transfection efficiency was analysed, and other tests were performed. The experiments were performed in triplicate.

### Detection of cell viability

Cell viability was determined by MTT assays. First, HASMCs were seeded in 96-well plates with a density of 4 × 10^4^ cells/well. After transfection for 48 h, 20 μL of MTT (Sigma, USA) was added to each well. The cells were incubated at 37 °C for 4 h. Then, 200 μL of DMSO (Sigma, USA) were added to each well to terminate the reaction. The optical density (OD) values were detected at 450 nm by using a microplate analyser (Bio-Rad, USA). The experiments were performed in triplicate.

Cell apoptosis was detected by performing flow cytometric analysis. Annexin V Alexa Fluor 488/PI Apoptosis Detection kit was purchased from Suolaibao (Beijing, China). A total of 5 × 10^5^ cells/well were seeded in 6-well plates and transfected after 48 h. The HASMCs were collected, and binding buffer was added to adjust the cell concentration to 1 × 10^6^ cells/mL. Then, 100 µL of the HASMC suspension was mixed with 5 μL of Annexin V-FITC and incubated at room temperature for 15 min in the dark. Then, 5 μL of propidium iodide were added to the cells and incubated in the dark for 5 min. Finally, cell apoptosis was determined by conducting flow cytometry (Beton Dickinson, USA). The experiments were performed in triplicate.

Cell necrocytosis was analysed using LDH Release Assay kits (Tiangen, China). The HASMCs were seeded in 96-well plates with a density of 4 × 10^4^ cells/well. After transfection for 48 h, the supernatant of the medium was collected. Then, 10 µL of the supernatant was mixed with NAD^+^/INT /diaphorase and incubated at room temperature for 30 min. Finally, the OD values were detected at 490 nm by using a microplate analyser (Bio-Rad, USA). The experiments were performed in triplicate.

### Luciferase reporter assays

The wild-type (WT) 3′-UTRs of COL1A1 and COL1A2 were amplified from the full-length cDNA by PCR. Then, COL1A1 3′-UTR and COL1A2 3′-UTR were digested with *Xho*I/*Bamh*I and inserted into the *Xho*I/*Bamh*I sites of the psiCHECK-2 vector (Promega, USA) to obtain the 3′-UTR-WT luciferase reporter plasmid. The mutant (MUT) 3′-UTR of COL1A1 and COL1A2 were synthesised (Sangon Biotech, China) and inserted into the psiCHECK-2 vector to obtain the 3′-UTR-MUT luciferase reporter plasmid.

The HASMCs were seeded in 24-well plates with a density of 1 × 10^5^ cells/well. When the cells grew to 60–70% confluence, they were co-transfected with the WT or MUT 3′-UTR reporter plasmid (50 ng) and 20 nM of the miR-513b-5p mimic or mimic-negative control by Lipofectamine 2000 Transfection Reagent. After transfection for 48 h, Firefly and Renilla luciferase activities were detected according to dual luciferase reporter assay (Promega, USA). GloMax 20/20 Luminometer (Promega, USA) was used to detect the luciferase activity. The relative luciferase activity was calculated by Firefly luciferase activity divided by the Renilla luciferase values. The experiments were performed in triplicate.

### SDS-PAGE and Western blot analysis

The protein samples were extracted from the cells by using RIPA buffer with PMSF (Beyotime, China). The protein concentrations were determined using the BCA Protein Assay Kit (Solaibao, China). Then, 20 μg of each protein sample was separated by 10% SDS–polyacrylamide gel and transferred to a pure nitrocellulose membrane (BioTrace, USA). The membrane was blocked with 5% bovine serum albumin (Beyotime, China) for 2 h and incubated with primary antibodies of COL1A2 (129 kD), TNF-α (25 kD), RIP3 (phospho S232, 53 kD), MLKL (phospho S358, 54 kD), caspase-8 (55 kD), caspase-8 (phospho S347, 55 kD), α-SMA (42 kD), collagen I (139 kD), collagen III (150 kD), MMP2 (74 kD), MMP3 (50 kD), MMP9 (92 kD), TIMP4 (25 kD) (1:1500, Abcam, UK), COL1A1 (1:1500, 220 kD), RIP 1 (1:1000, 78 kD), RIP (phospho S166) (1:1000, 78–82 kD), and GAPDH (1:3000, 37 kD) (Cell Signaling Technology, USA) at 4 °C overnight. The following day, the membranes were washed thrice with Tris-buffered saline containing 0.1% Tween-20 (Beyotime, China) and incubated with an anti-mouse HRP-conjugated secondary antibody (1:5000, Abcam, UK) for 2 h. Then, the chemiluminescence signals were detected using the ECL Western blot detection kit (Thermo, USA). The bands were collected by the GEL SensiCapture imaging system (Peiqing Technology Co. LTD, China) and analysed with Image J. The experiments were performed in triplicate.

### Statistical analysis

All experiments were analysed using SPSS 20.0 (IBM, USA). For the normally distributed data, Student’s *t *test was performed to compare two groups and one-way ANOVA with LSD post hoc test for more than two groups. For non-normally distributed data, Mann–Whitney *U* test was used to compare two groups and Kruskal–Wallis H (K) for more than two groups. Counting data were analysed by Chi-square test. *p* < 0.05 was used to consider statistical significance.

## Results

### Participant characteristics

The participants (control [n = 100], un-ruptured IA [n = 100] and ruptured IA [n = 100]) did not differ in age, gender, total cholesterol level, low-density lipoprotein cholesterol level, hypertension, diabetes mellitus, coronary heart disease, cerebral infarction, hypertension with diabetes mellitus, smoking status and drinking status. However, the C-reactive protein, high-density lipoprotein cholesterol, fasting blood glucose, triglyceride, uric acid, glycosylated haemoglobin, creatinine, total plasma homocysteine, brain natriuretic peptide, Hunt Hess Grade, Glasgow Coma score, World Federation of Neurosurgical Societies Grade and modified Ranking scale were different amongst the three groups (Table 2).

**Table 2:** Participants’ clinical characteristics.

Factors	Control (n = 100)	Unrupture IA (n = 100)	Rupture IA (n = 100)	*p* value
Age (years)	60 (53–66)	59 (50–67)	55.5 (49.25–62.75)	0.123^a^
Men	57 (57%)	53 (53%)	50 (50%)	0.609^b^
CRP (mg/L)	2.81 (1.6–7.09)	4 (2.22–8.37)*	8.64 (3–19.47)*^,#^	< 0.001^a^
TC (mmol/L)	4.42 ± 0.12	4.33 ± 0.11	4.35 ± 0.11	0.851^c^
LDL-C (mmol/L)	2.99 ± 0.10	2.68 ± 0.07	2.81 ± 0.10	0.077^c^
HDL-C (mmol/L)	1.06 (0.87–1.29)	1.19 (0.88–1.56)*	1.19 (0.96–1.48) *	0.027^a^
GLU (mmol/L)	5.36 (4.88–6.66)	4.98 (4.46–5.63)*	6.29 (5.31–7.56)*^,#^	< 0.00^a^
TG (mmol/L)	1.27 (0.91–1.83)	1.46 (0.91–1.75)	1.08 (0.76–1.48)*^,#^	0.021^a^
UA (mmol/L)	356 (255–405.75)	304 (232–369.75)*	242 (163.75–323)*^,#^	< 0.001^a^
HbA1c (mmol/L)	6.1 (5.6–7.5)	6 (5.6–6.67)	5.7 (5.5–6)*^,#^	< 0.001^a^
Creatinine (μmol/L)	70 (58–81.75)	65 (57–79)*	60 (50–71)*^,#^	< 0.001^a^
Hcy (mmol/L)	8.2 (9.85–13.8)	8.05 (6.25–10.17)*	9.32 (7.8–13.07)^#^	< 0.001^a^
BNP (pg/mL)	91.14 (45.08–180.11)	77.09 (42.01–113.09)*	180 (61.48–578.95)*^,#^	< 0.001^a^
Hypertension	42 (42%)	40 (40%)	44 (44%)	0.849^b^
Diabetes mellitus	14 (14%)	19 (19%)	14 (14%)	0.532^b^
Coronary heart disease	7 (7%)	11 (11%)	8 (8%)	0.578^b^
cerebral infarction	15 (15%)	14 (14%)	18 (18%)	0.720^b^
Hypertension + Diabetes mellitus	18 (18%)	11 (11%)	13 (13%)	0.340^b^
Others	4 (4%)	5 (5%)	3 (3%)	0.769^b^
**Smoking status**				0.697^b^
Never	30 (30%)	23 (23%)	28 (28%)	
Current	26 (26%)	33 (33%)	29 (29%)	
Previously	21 (21%)	25 (25%)	27 (27%)	
Occasionally	23 (23%)	19 (19%)	16 (16%)	
**Alcohol drinking**				0.528^b^
Never	22 (22%)	28 (28%)	25 (25%)	
≤ 3 times/week	33 (33%)	34 (34%)	34 (34%)	
> 4 times/week	15 (15%)	17 (17%)	22 (22%)	
Occasionally	30 (30%)	21 (21%)	19 (19%)	
Hunt Hess Grade	0 (0–0)	0 (0–0)*	2 (1–3)*^,#^	< 0.001^a^
GCS Grade	0 (0–0)	15 (15–15)*	15 (14–15)*^,#^	< 0.001^a^
WFNS Grade	0 (0–0)	1 (1–1)*	1 (1–2)*^,#^	< 0.001^a^
mRS	0 (0–0)	0 (0–0)*	0 (0–2)*^,#^	< 0.001^a^

IA, intracranial aneurysm; CRP, C-reactive protein; TC, total cholesterol; LDL-C, low-density lipoprotein cholesterol; HDL-C, high-density lipoprotein cholesterol; GLU, Fasting blood glucose; TG, Triglyceride; UA, Uric acid; HbA1c, Glycosylated hemoglobin; Hcy, Total plasma homocysteine; BNP, brain natriuretic peptide; GCS, Glasgow Coma score; WFNS, World Federation of Neurosurgical Societies; mRS, modified Ranking scale. Data are presented as mean ± standard error, the number and percentage (%) and median with interquartile interval [Q1–Q3]. Compared with control group, *p  < 0.05; Compared with UA group, ^#^*p* < 0.05, Mann–Whitney *U* test with two-sided. a, Kruskal–Wallis H (K) test; b, Chi-square test; c, one-Way ANOVA analysis of variance with LSD test. *P* < 0.05 was considered to suggest a significant difference.

The characteristics of IA before treatment showed that the aneurysm was mainly located in the anterior circulation, with 81% un-ruptured IA and 90% ruptured IA, followed by the posterior and cerebral circulations. No significant differences were found in the distribution of the location of aneurysm. The main morphologies of the un-ruptured IA were dissecting aneurysm (32%), followed by multiple, seed tumour and lobular. Seed tumour (27%) was the main morphology of the ruptured IA, followed by multiple, dissecting aneurysm and lobular. Significant differences were found in the aneurysm morphologies (*p* = 0.034). No significant differences were found in the length, depth and maximal diameter of the aneurysm between the un-ruptured and rupture IAs. However, the short diameter and neck size in the un-ruptured IA were significantly higher than those in the ruptured IA (*p* = 0.044 and *p* = 0.004, respectively) (Table 3).

**Table 3:** Intracranial aneurysm characteristics.

Characteristics	Unrupture IA (n = 100)	Rupture IA (n = 100)	*p* value
**Aneurysm location n (%)**			0.173^a^
Anterior circulation	81 (81%)	90 (90%)	
Posterior circulation	16 (16%)	9 (9%)	
Cerebral circulation	3 (3%)	1 (1%)	
**Aneurysm morphology n (%)**			0.034^a^
Multiple	17 (17%)	26 (26%)	
Dissecting aneurysm	32 (32%)	21 (21%)	
Lobular	16 (16%)	18 (18%)	
Seed tumor	17 (17%)	27 (27%)	
Others	18 (18%)	8 (8%)	
**Aneurysm size (mm)**			
Length	6.99 (5.19–9.08)	6.14 (4.74–8.21)	0.546^b^
Depth	4.83 (3.25–6.61)	4.96 (3.34–6.28)	0.566^b^
Short diameter	4.48 (3.27–6.54)	3.50 (2.69–5.45)	0.044^b^
Neck size	3.77 (2.47–6.60)	3.20 (2.28–3.99)	0.004^b^
Maximal diameter	2.78 (2.07–3.75)	2.75 (1.96–3.51)	0.479^b^

IA, intracranial aneurysm. Data are presented as the number and percentage (%), and median with interquartile interval [Q1–Q3]. a, Chi-square test; b, Mann–Whitney test. *p* < 0.05 was considered to suggest a significant difference.

### Serum miR-513b-5p and related gene expression

**Figure 1:**
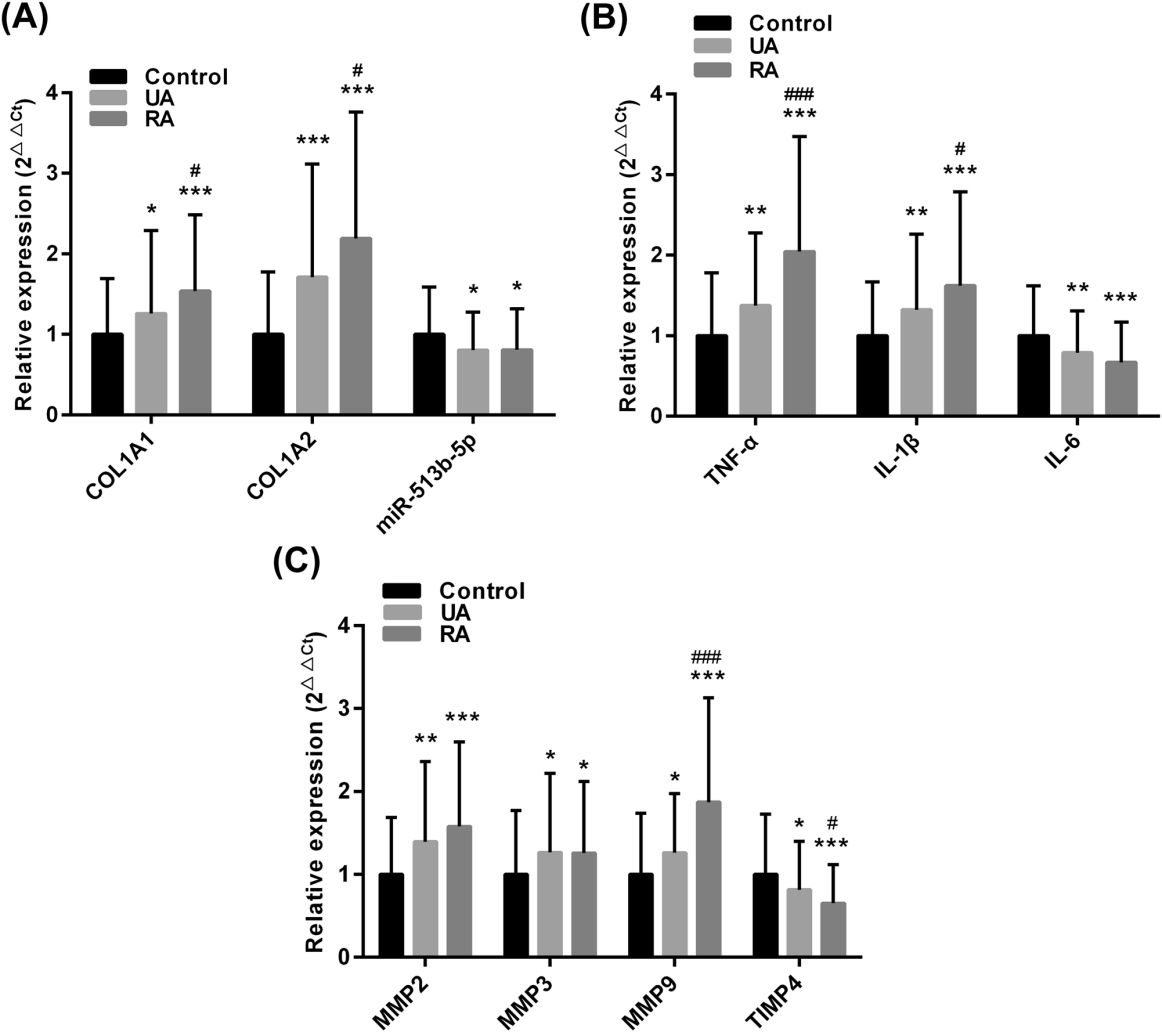
Serum miR-513b-5p and related gene expression in participants. Quantitative RT-PCR assay was used to detected gene expression. (**A**) miR-513b-5p, COL1A1 and COL1A2 expression. (**B**) Inflammatory cytokine of TNF-α, IL-1β and IL-6 mRNA expression. (**C**) MMP2, MMP3, MMP9 and TIMP4 mRNA expression. UA, unrupture aneurysm (n = 100); RA, rupture aneurysm (n = 100); control (n = 100). MiR-513b-5p expression was normalized to cel-miR-39, other mRNA expression was normalized to GAPDH. All of the data are presented as the mean ± standard deviation. Compared with control group, *p < 0.05; **p < 0.01 and ***p < 0.001. Compared with UA group, ^#^*p* < 0.05 and ^###^*p* < 0.001. Mann–Whitney *U* test with two-sided was performed.

The mRNA levels of COL1A1, COL1A2, TNF-α, IL-1β, MMP2, MMP3 and MMP9 were significantly increased in patients with IA compared with the control group (*p* < 0.05). The expression of miR-513b-5p, IL-6 and TIMP4 was significantly decreased in the patients with IA compared with the control group (*p* < 0.05). The mRNA levels of COL1A1, COL1A2, TNF-α, IL-1β and MMP9 were significantly higher in the ruptured IA than the un-ruptured type (*p* < 0.05). However, TIMP4 expression in the un-ruptured IA was significantly lower than that in the ruptured IA (*p* < 0.05) (Fig. 1).

### MiR-513b-5p targeted the inhibition of the expression of COL1A1 and COL1A2

**Figure 2:**
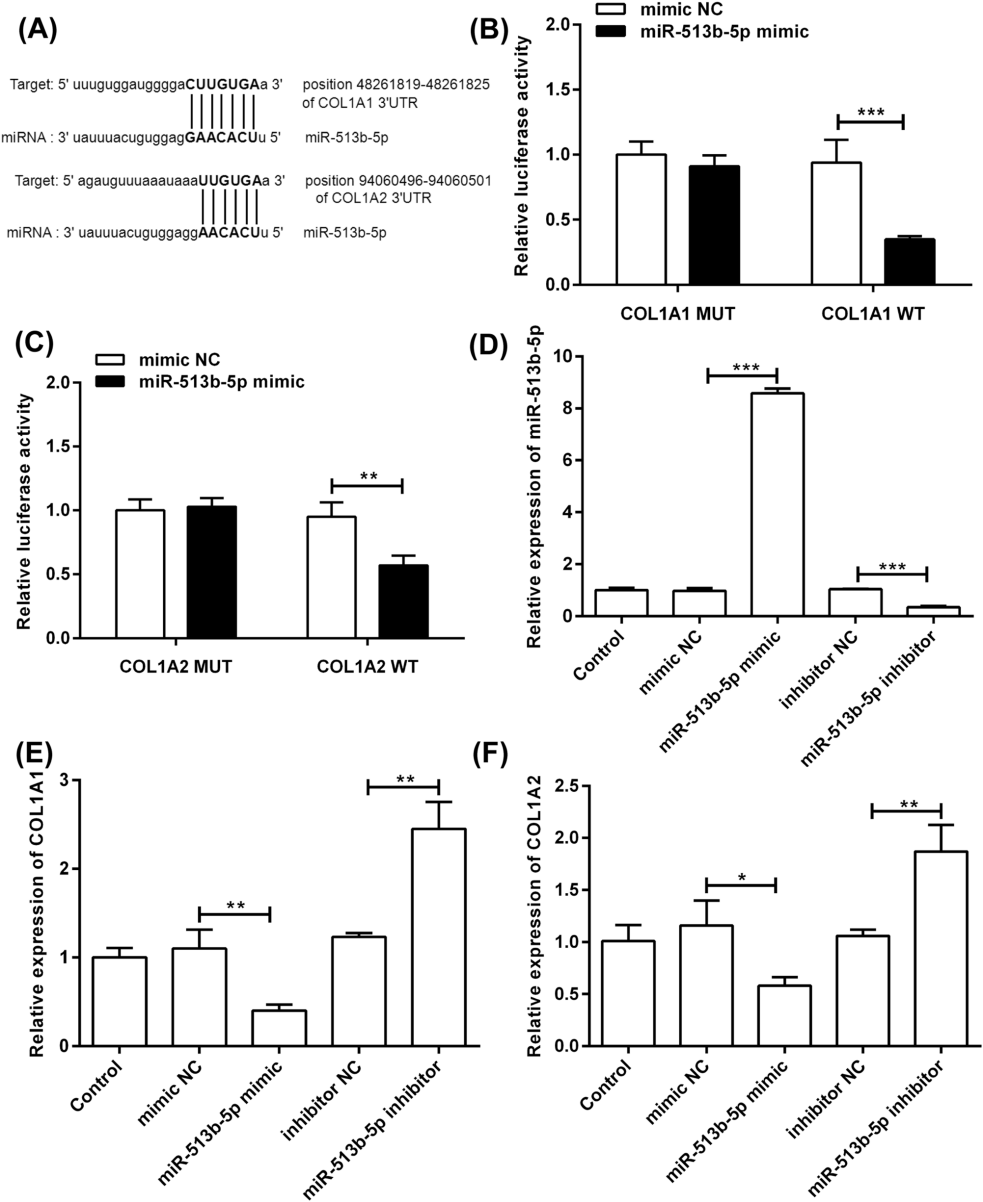
MiR-513b-5p directly targeted COL1A1 and COL1A2 and inhibited their expression. (**A**) Schematic representation of miR-513b-5p binding sites in COL1A1-3′UTR and COL1A2-3′UTR. (**B**) The dual luciferase report assay confirmed the targeting relationship between miR-513b-5p and COL1A1-3′UTR. (**C**) The targeting relationship between miR-513b-5p and COL1A2-3′UTR was proved by the dual luciferase report assay. (**D**) The miR-513b-5p mimic and inhibitor transfected with the HASMCs. (**E**) MiR-513b-5p inhibited the COL1A1 mRNA expression. (F) MiR-513b-5p inhibited the COL1A2 mRNA expression. All data presented as mean ± standard deviation, and three independent experiments were carried out. Compared with mimic NC or inhibitor NC group, *p < 0.05, **p < 0.01, and ***p < 0.001. Student's *t *test with two-sided was performed.

The results from Targetscan (http://www.targetscan.org/) and Starbase (http://starbase.sysu.edu.cn/) showed that miR-513b-5p had binding sites with COL1A1-3′UTR and COL1A2-3′UTR (Fig. 2A). COL1A1 and COL1A2 may be the target genes of miR-513b-5p. Then, in the HASMCs, a dual luciferase reporter assay was used to demonstrate the targeting relationship between miR-513b-5p and COL1A1-3′UTR or COL1A2-3′UTR. The results showed that co-transfection with miR-513b-5p mimic and reporter plasmid of COL1A1-3′UTR WT or COL1A2-3′UTR WT significantly decreased the luciferase activity, contrary to the result obtained using COL1A1-3′UTR MUT (Fig. 2B) or COL1A2-3′UTR MUT (Fig. 2C). This result indicated that miR-513b-5p regulated the expression of COL1A1 and COL1A2 by binding to the target sites.

In addition, transfection of the miR-513b-5p mimic significantly induced the miR-513b-5p expression compared with mimic NC, and the miR-513b-5p inhibitor significantly reduced the miR-513b-5p expression compared with the inhibitor NC (Fig. 2D). Compared with the mimic NC, the miR-513b-5p mimic significantly reduced the mRNA expression of COL1A1 (Fig. 2E) and COL1A2 (Fig. 2F). However, the mRNA expression of COL1A1 and COL1A2 was significantly increased by the miR-513b-5p inhibitor compared with the inhibitor NC. These results suggested that miR-513b-5p directly targeted COL1A1 and COL1A2 and suppressed their expression.

### MiR-513b-5p regulated the viability of the HASMC by the RIP1-RIP3-MLKL and MMP pathways

**Figure 3:**
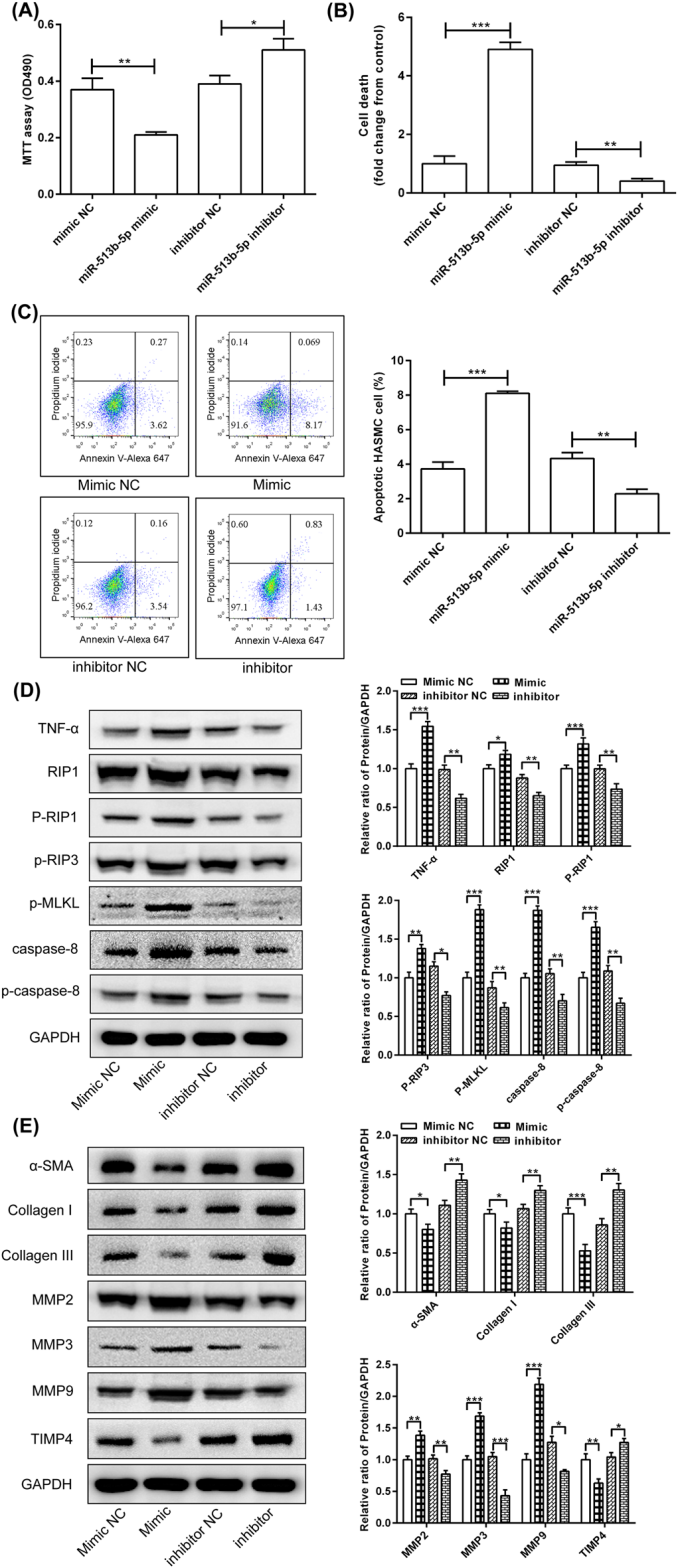
MiR-513b-5p regulated the viability of the HASMCs by the RIP1-RIP3-MLKL and MMP pathways. (**A**) MTT assay was used to analyse HASMC cell viability. (**B**) LDH release assay was used to detect cell death. (**C**) Flow cytometric analysis was performed to detect cell apoptosis. (**D**) Western blot was used to detect the expression of related proteins in the RIP1-RIP3-MLKL pathway. (**E**) Analyze the expression of related proteins in the MMP pathway. All data presented as the mean ± standard deviation, and three independent experiments were carried out. Compared with mimic NC or inhibitor NC group, *p < 0.05, **p < 0.01, and ***p < 0.001. Student's *t *test with two-sided was performed.

To determine the effect of miR-513b-5p on the biological behaviour of the HASMCs, the cell proliferation, death and apoptosis were tested by transfecting miR-513b-5p mimic or inhibitor. The MTT results showed that the miR-513b-5p mimic significantly inhibited the proliferation of the HASMCs, whereas miR-513b-5p inhibitor significantly enhanced cell proliferation (Fig. 3A). The results from the LDH release assay revealed that transfection of the miR-513b-5p mimic significantly induced cell death, but the miR-513b-5p inhibitor significantly decreased cell death (Fig. 3B). Flow cytometry was performed to detect cell apoptosis, and the results exhibited that cell apoptosis was promoted by the miR-513b-5p mimic, contrary to the effect of the miR-513b-5p inhibitor (Fig. 3C).

To further determine the effects of miR-513b-5p on the death, apoptosis and plaque instability of the HASMCs, Western blot was used to analyse the RIP1-RIP3-MLKL and MMP pathways. Compared with the mimic NC, the miR-513b-5p mimic significantly increased the protein expression of TNF-α, RIP1, phospho-RIP1, phospho-RIP3, phospho-MLKL, caspase-8 and phospho-caspase-8. By contrast, compared with inhibitor NC, the miR-513b-5p inhibitor significantly decreased the expression of these proteins (Fig. 3D). In addition, compared with those in the mimic NC group, the protein levels of α-SMA, collagen I, collagen III and TIMP4 were significantly inhibited by the miR-513b-5p mimic but MMP2, MMP3 and MMP9 were significantly increased. Compared with the inhibitor NC, the miR-513b-5p inhibitor significantly induced the expression of α-SMA, collagen I, collagen III and TIMP4 but significantly decreased MMP2, MMP3 and MMP9 (Fig. 3E).

### COL1A1 rescued the effect of miR-513b-5p on the viability of the HASMCs by the RIP1-RIP3-MLKL and MMP pathways

**Figure 4:**
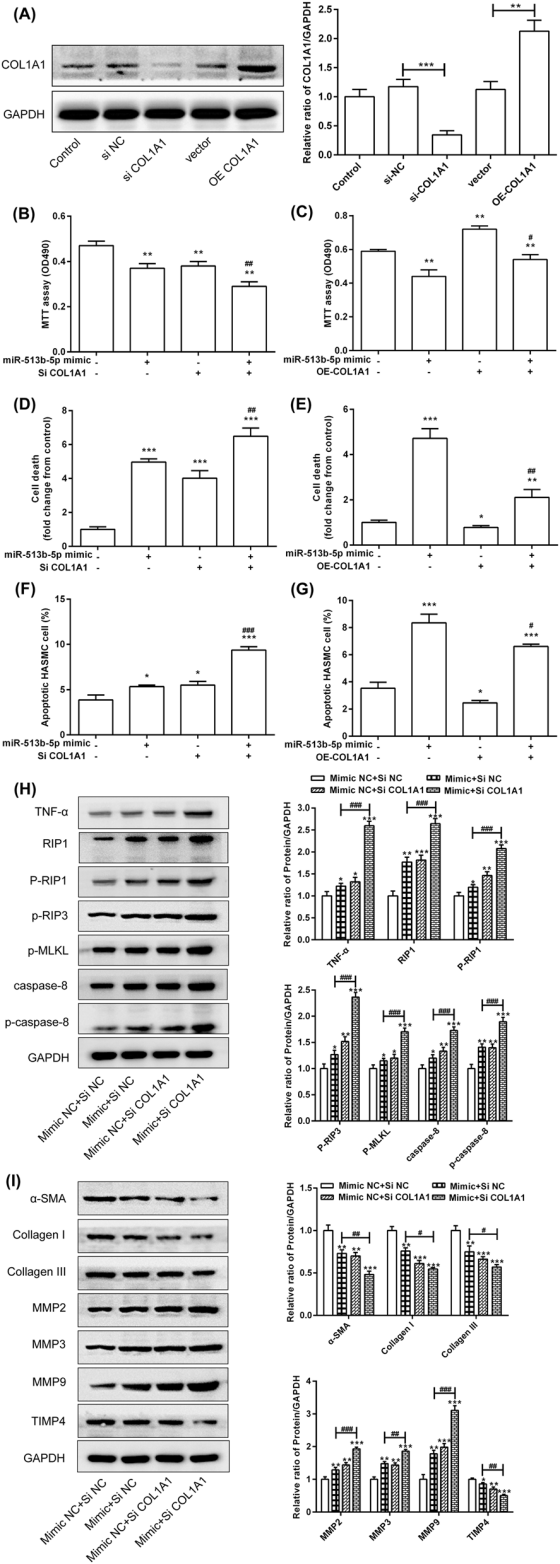
COL1A1 rescued the effect of miR-513b-5p on viability of HASMCs by the RIP1-RIP3-MLKL and MMP pathways. (**A**) The protein and mRNA expression was detected after COL1A1 interference and over-expression. (**B**) Interference with COL1A1 enhanced the inhibitory effect of miR-513b-5p on the cell proliferation. (**C**) Over-expression of COL1A1 inhibited the inhibitory effect of miR-513b-5p on the cell proliferation. (**D**) Interference with COL1A1 enhanced the induction of cell death by miR-513b-5p. (**E**) Over-expression of COL1A1 inhibited the induction of cell death by miR-513b-5p. (**F**) Interference with COL1A1 enhanced the induction of cell apoptosis by miR-513b-5p. (**G**) Over-expression of COL1A1 inhibited the induction of cell apoptosis by miR-513b-5p. COL1A1 regulated the expression of related proteins in the (**H**) RIP1-RIP3-MLKL and (**I**) MMP pathways. All data presented as the mean ± standard deviation, and three independent experiments were carried out. Compared with mimic NC + siRNA NC group, *p < 0.05, **p < 0.01, and ***p < 0.001. Compared with miR-513b-5p mimic + siRNA NC group, ^#^*p* < 0.05, ^##^*p* < 0.01, and ^###^*p* < 0.001. One-way ANOVA with two-sided LSD post hoc test was performed.

To clarify the exact role of COL1A1 in the miR-513b-5p-regulated cell proliferation, death, apoptosis and extracellular matrix formation, the *COL1A1* gene was over-expressed or inhibited in the HASMCs. Interference of COL1A1 significantly inhibited the protein and mRNA expression of COL1A1, whilst over-expression of COL1A1 significantly promoted the protein and mRNA expression of COL1A1 (Fig. 4A). Interference with COL1A1 significantly reduced the proliferation of the HASMCs and enhanced the effect of miR-513b-5p mimic in inhibiting cell proliferation (Fig. 4B). Over-expression of COL1A1 significantly induced the proliferation of the HASMCs and improved the effect of the miR-513b-5p mimic in inhibiting cell proliferation (Fig. 4C). Down-regulation of COL1A1 significantly increased cell death and enhanced the effect of miR-513b-5p-induced cell death (Fig. 4D). By contrast, the over-expression of COL1A1 significantly decreased cell death and reduced the miR-513b-5p-induced cell death (Fig. 4e). In addition, down-regulation of COL1A1 significantly promoted cell apoptosis and enhanced the effect of the miR-513b-5p-induced cell apoptosis (Fig. 4F). However, over-expression of COL1A1 significantly inhibited cell apoptosis and decreased the miR-513b-5p-induced cell apoptosis (Fig. 4G).

The results of Western blot showed that inhibiting COL1A1 expression significantly increased the protein expression of TNF-α, RIP1, phospho-RIP1, phospho-RIP3, phospho-MLKL, caspase-8 and phospho-caspase-8 and enhanced the miR-513b-5p-induced expression of these proteins (Fig. 4H). However, inhibiting COL1A1 significantly decreased the expression of α-SMA, collagen I, collagen III and TIMP4 but significantly increased MMP2, MMP3 and MMP9 (Fig. 4I). Co-transfection with the miR-513b-5p mimic and COL1A1 siRNA enhanced the inhibition of miR-513b-5p on α-SMA, collagen I, collagen III and TIMP4 expression and promoted the induction of miR-513b-5p on MMP2, MMP3 and MMP9 expression (Fig. 4I).

### COL1A2 rescued the effect of miR-513b-5p on the viability of HASMCs by the RIP1-RIP3-MLKL and MMP pathways

**Figure 5:**
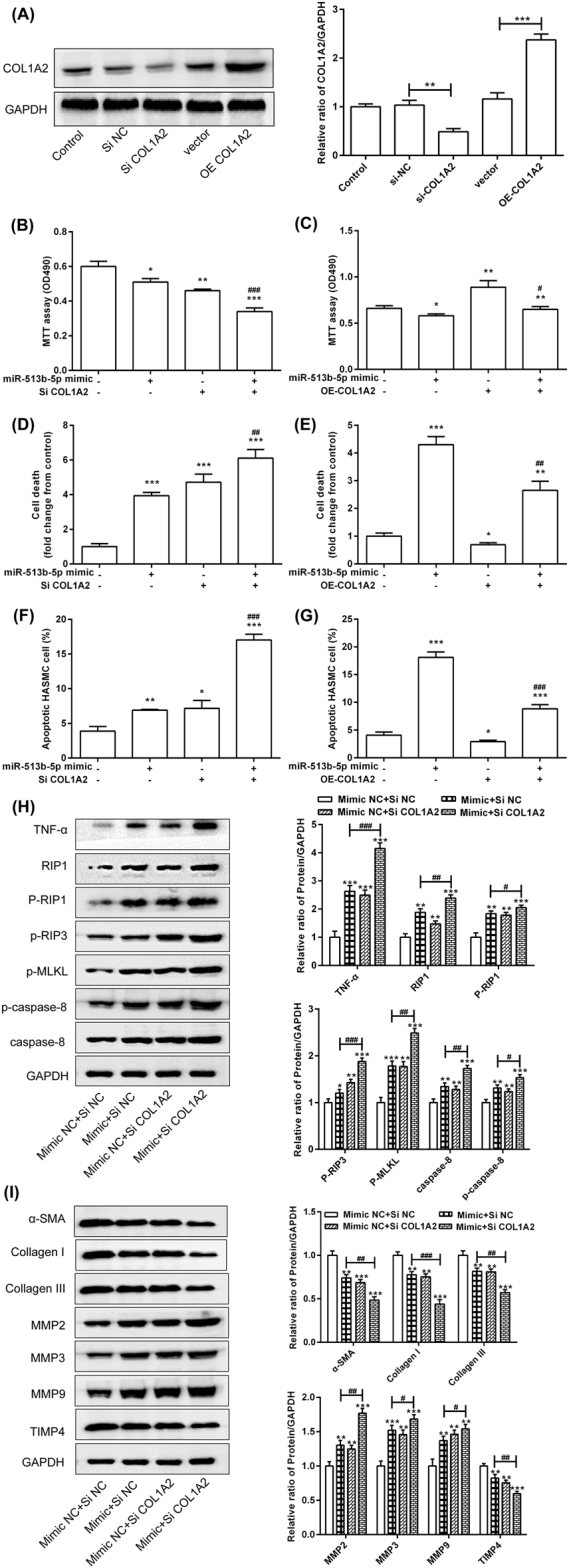
COL1A2 rescued the effect of miR-513b-5p on viability of HASMC by the RIP1-RIP3-MLKL and MMP pathways. (**A**) The protein and mRNA expression was detected after COL1A2 interference and over-expression. (**B**) Interference with COL1A2 enhanced the inhibitory effect of miR-513b-5p on the cell proliferation. (**C**) Over-expression of COL1A2 inhibited the inhibitory effect of miR-513b-5p on cell proliferation. (**D**) Interference with COL1A2 enhanced the induction of cell death by miR-513b-5p. (**E**) Over-expression of COL1A2 inhibited the induction of cell death by miR-513b-5p. (**F**) Interference with COL1A2 enhanced the induction of cell apoptosis by miR-513b-5p. (**G**) Over-expression of COL1A2 inhibited the induction of cell apoptosis by miR-513b-5p. COL1A2 regulated the expression of related proteins in the (**H**) RIP1-RIP3-MLKL pathway and (**I**) MMP pathway. All data were expressed as the mean ± standard deviation, and three independent experiments were carried out. Compared with mimic NC + siRNA NC group, *p < 0.05, **p < 0.01, and ***p < 0.001. Compared with miR-513b-5p mimic + siRNA NC group, ^#^*p* < 0.05, ^##^*p* < 0.01, and ^###^*p* < 0.001. One-way ANOVA with two-sided LSD post hoc test was performed.

The exact roles of COL1A2 in the miR-513b-5p-regulated cell proliferation, death, apoptosis and extracellular matrix formation were tested by the over-expression or inhibition of COL1A2 in the HASMCs. First, the expression of COL1A2 was interfered and over-expressed (Fig. 5A). Interference with COL1A2 significantly enhanced the inhibitory effect of miR-513b-5p on cell proliferation (Fig. 5B). Over-expression of COL1A2 significantly improved the inhibitory effect of miR-513b-5p mimic on proliferation (Fig. 5C). Down-regulation of COL1A2 significantly enhanced the induction of cell death by miR-513b-5p (Fig. 5D), but over-expression of COL1A2 significantly reduced the induction of cell death by miR-513b-5p (Fig. 5E). In addition, interference with COL1A2 significantly enhanced the induction of cell apoptosis by miR-513b-5p (Fig. 5F), but over-expression of COL1A2 significantly decreased this cell apoptosis (Fig. 5G).

In addition, inhibition of the expression of COL1A2 significantly increased the protein expression of TNF-α, RIP1, phospho-RIP1, phospho-RIP3, phospho-MLKL, caspase-8 and phospho-caspase-8, which was induced by miR-513b-5p (Fig. 5H). Then, COL1A2 knockdown significantly enhanced the inhibition of miR-513b-5p on the expression of α-SMA, collagen I, collagen III and TIMP4 and significantly increased that of MMP2, MMP3 and MMP9 induced by miR-513b-5p (Fig. 5I). These results revealed that the role of COL1A1 and COL1A2 may play similar roles in the HASMCs.

### MiR-513b-5p/COL1A1 affected the vitality of the HASMCs during TNF-α intervention by regulating the RIP1-RIP3-MLKL and MMP pathways

**Figure 6:**
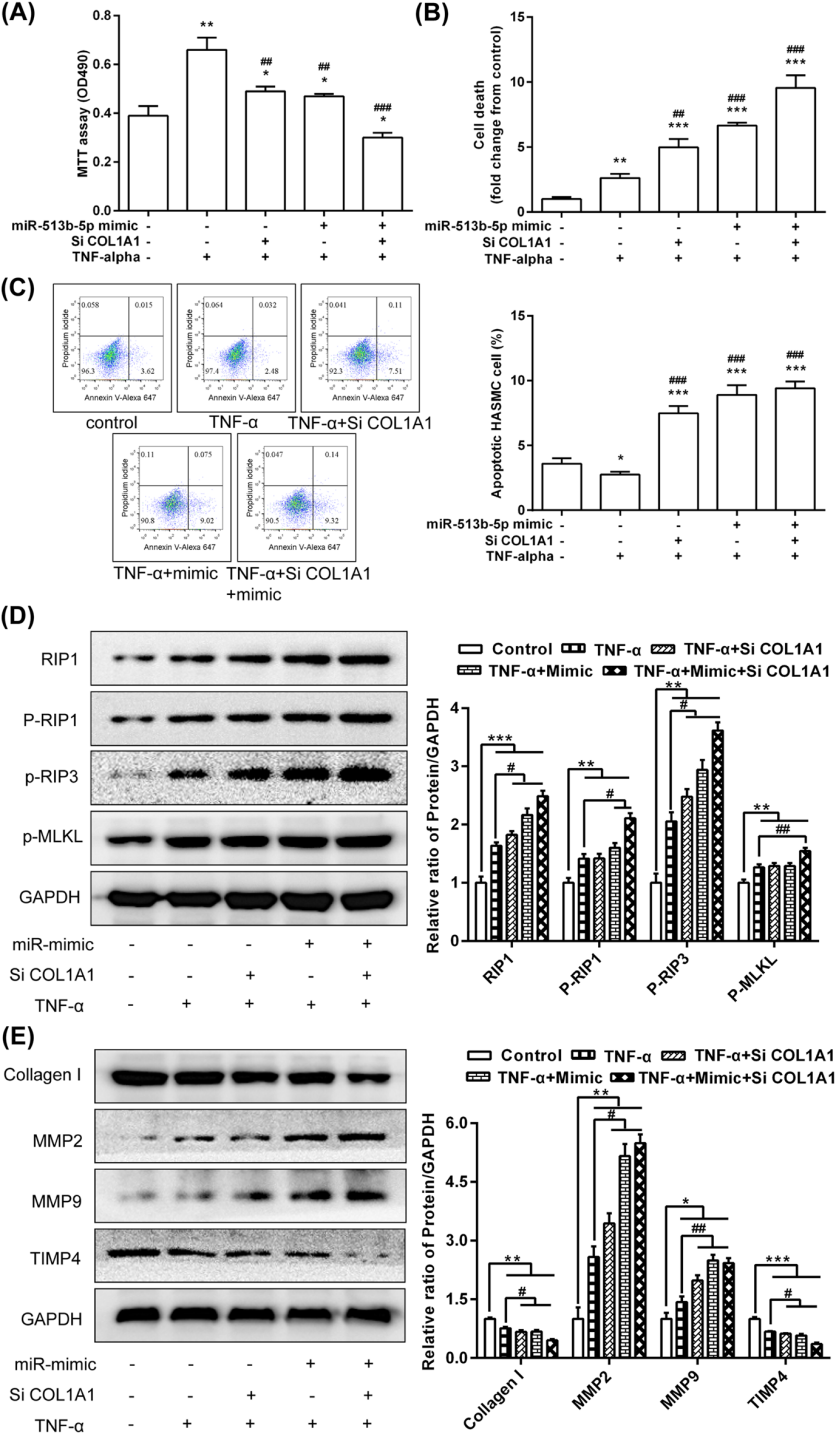
MiR-513b-5p/COL1A1 affected the vitality of the HASMCs during TNF-α intervention by RIP1-RIP3-MLKL and MMPs pathways. (**A**) MTT assay was used to analyse cell viability. (**B**) LDH release assay was used to detect cell death. (**C**) Flow cytometric analysis was performed to detect cell apoptosis. (**D**) Western blot was used to detect the expression of RIP1-RIP3-MLKL pathway. (**E**) The expression of related proteins in the MMP pathway. All data are expressed as means ± standard deviation, and three independent experiments were carried out. Compared with mimic NC + siRNA NC + TNF-α (−) group, *p < 0.05, **p < 0.01, and ***p < 0.001. Compared with mimic NC + siRNA NC + TNF-α (+) group, ^#^*p* < 0.05, ^##^*p* < 0.01, and ^###^*p* < 0.001. One-way ANOVA with two-sided LSD post hoc test was performed.

TNF-α could induce HASMC necroptosis, extracellular matrix degradation, and remodelling, which increased the fragility of the blood vessel walls and outward expansion of the arterial walls, thereby promoting the formation and even rupture of IA. The roles of miR-513b-5P in targeting COL1A1 were analysed in the TNF-α. First, TNF-α induced the proliferation of the HASMCs, but miR-513b-5p mimic and/or inhibition of expression of COL1A1 significantly decreased the induction effect of TNF-α on cell proliferation (Fig. 6A). Second, TNF-α induced the death of the HASMCs, and miR-513b-5p mimic and/or COL1A1 siRNA significantly enhanced the induction effect of TNF-α on cell death (Fig. 6B). Third, TNF-α inhibited the apoptosis of the HASMCs, whilst miR-513b-5p mimic and/or COL1A1 siRNA significantly promoted the apoptosis of TNF-α intervention (Fig. 6C).

The Western blot results revealed that TNF-α significantly induced the protein expression of RIP1, phospho-RIP1, phospho-RIP3 and phospho-MLKL. Co-transfection of the miR-513b-5p mimic and COL1A1 siRNA significantly enhanced the induction of these proteins by TNF-α (Fig. 6D). In addition, TNF-α treatment significantly induced the protein expression of MMP2 and MMP9. Transfection of miR-513b-5p mimic and/or COL1A1 siRNA significantly enhanced the induction of the proteins of MMP2 and MMP9 by TNF-α (Fig. 6E). However, the expression of collagen I and TIMP4 were significantly decreased after TNF-α treatment. Transfection of miR-513b-5p mimic and/or COL1A1 siRNA enhanced the inhibition of collagen I and TIMP4 proteins by TNF-α (Fig. 6E).

### MiR-513b-5p/COL1A2 affected the vitality of HASMCs during TNF-α intervention by regulating the RIP1-RIP3-MLKL and MMP pathways

**Figure 7:**
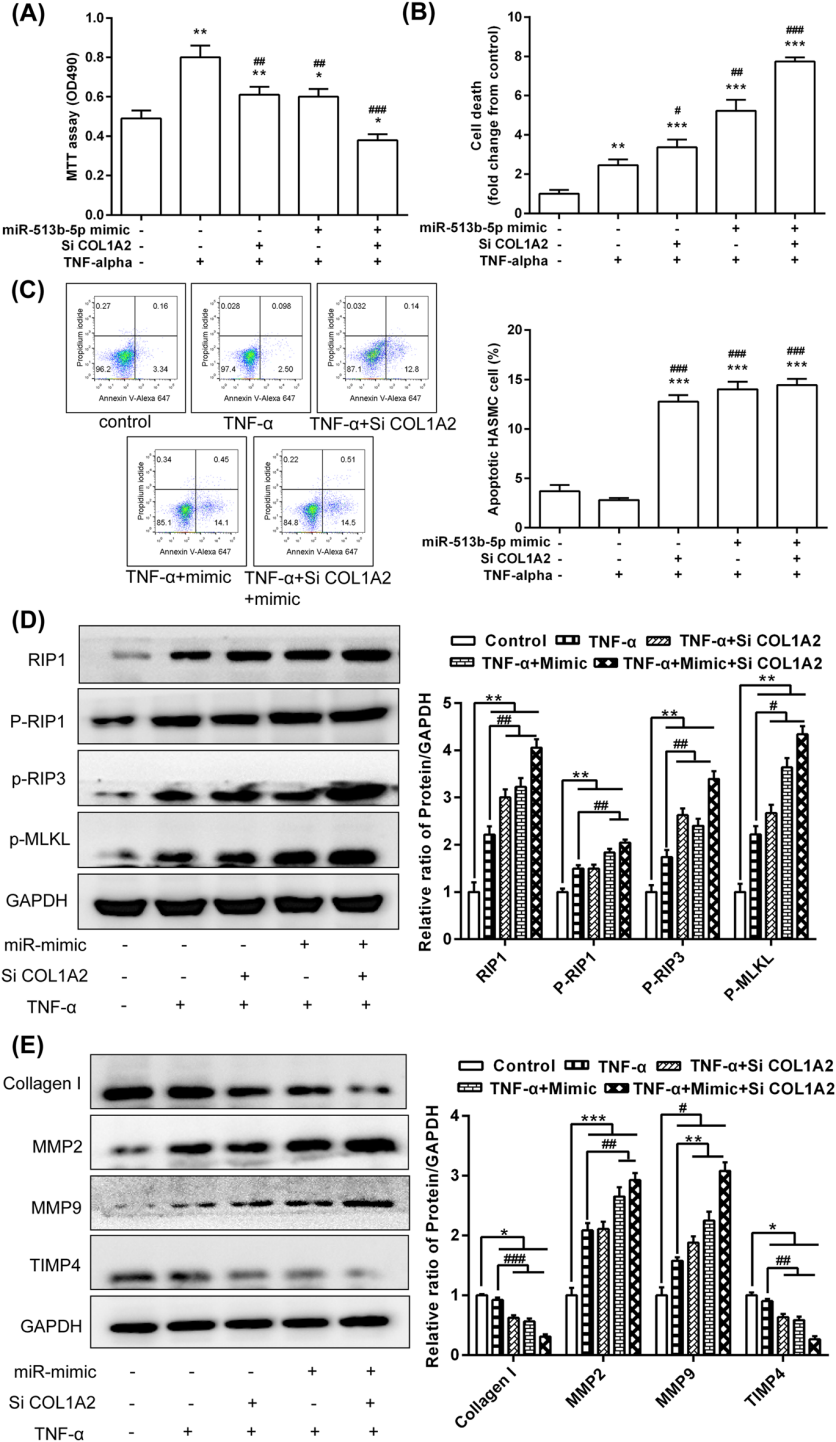
MiR-513b-5p/COL1A2 affected the vitality of the HASMCs during TNF-α intervention by RIP1-RIP3-MLKL and MMPs pathways. (**A**) MTT assay was used to analyse cell viability. (**B**) LDH release assay was used to detect cell death. (**C**) Flow cytometric analysis was performed to detect cell apoptosis. (**D**) Western blot was used to detect the expression of RIP1-RIP3-MLKL pathway. (**E**) The expression of related proteins in the MMP pathway. All data are expressed as means ± standard deviation, and three independent experiments were carried out. Compared with mimic NC + siRNA NC + TNF-α (−) group, *p < 0.05, **p < 0.01, and ***p < 0.001. Compared with mimic NC + siRNA NC + TNF-α (+) group, ^#^*p* < 0.05, ^##^*p* < 0.01, and ^###^*p* < 0.001. One-way ANOVA with two-sided LSD post hoc test was performed.

The results showed that the miR-513b-5p mimic and/or COL1A1 siRNA significantly decreased the induction effect of TNF-α on the proliferation of HASMCs (Fig. 7A) but significantly enhanced the induction effect of TNF-α on cell death (Fig. 7B) and apoptosis (Fig. 7C). The effect of TNF-α-induced protein expression of RIP1, phospho-RIP1, phospho-RIP3 and phospho-MLKL was enhanced by the miR-513b-5p mimic and/or COL1A1 siRNA (Fig. 7D). TNF-α-induced protein expression of MMP2 and MMP9 was significantly enhanced by the miR-513b-5p mimic and/or COL1A1 siRNA, but the expression of collagen I and TIMP4 was significantly inhibited (Fig. 7E).

## Discussion

IA is a disease of intracranial arterial dilatation. When the arterial wall is impacted by haemodynamic shear force and a series of pathophysiological changes, the arterial blood vessels will swell and protrude until an aneurysm is formed^3,5–7^. Some IAs are relatively stable due to special anatomical locations, whilst other IAs are due to the death or loss of parietal cells or degeneration of the extracellular matrix, which eventually leads to the rupture of the vascular wall^14,15^. Previous studies have shown that chronic inflammatory responses, loss of smooth muscle cells and degradation of the extracellular matrix are important characters of ruptured and un-ruptured aneurysms^2,27–30^.

The results showed that the mRNA expression of COL1A1, COL1A2, TNF-α, IL-1β, MMP2, MMP3 and MMP9 was significantly increased but IL-6 and TIMP4 were significantly decreased in patients with IA. Collagen I and collagen III are the most abundant collagen types in the arteries and are therefore important for the strength of arterial walls^31^. Increased expression of COL1A1 and COL1A2 may indicate abnormal collagen metabolism and structure incompleteness^17^. This phenomenon is consistent with the results of the collagen degradation caused by the increased activity of inflammatory factors TNF-α, IL-1β and MMP2, MMP3 and MMP9^24,32–36^. The growth of IA is characterised by the frailness of the vessel wall. The loss of internal elastic layer is one of the initial vascular reactions caused by aneurysm. Local matrix degradation is related to local cell apoptosis and increased expression of MMPs^37^. MMPs belong to a family of proteinases which participate in the remodelling of vascular walls. The increased activity of MMP2, MMP3 and MMP9 in the arterial walls contributes to the inflammatory response and extracellular matrix degeneration during IA^33,34^. TIMPs are endogenous inhibitors of MMPs. TIMP4 is known to be capable of inhibiting the activity of all MMPs^30^. Decreased expression of TIMP4 may lead to the increased activity of MMPs, resulting in uncontrolled extracellular matrix remodelling and subsequent development of IA. In addition, this study revealed that the expression of COL1A1, COL1A2, TNF-α, IL-1β and MMP9 was significantly increased in the patients with ruptured IA compared with those with un-ruptured IA. The expression of TIMP4 was significantly decreased. These results are consistent with the finding of Roder^17^, suggesting that inflammation and collagen I degeneration existed during IA.

The expression of miR-513b-5p was significantly decreased in patients with ruptured and non-ruptured IAs. The result further confirmed that miR-513b-5p targeting of COL1A1 and COL1A2 inhibited the proliferation of the HASMCs and promoted cell death and apoptosis by activating the RIP1-RIP3-MLKL and MMP pathways. Previous studies have shown that miR-513b-5p can inhibit cell proliferation and promote apoptosis, such as in testicular cells^38^ and ovarian cancer cells^26^. Inhibition of COL1A1 and COL1A2 could supress collagen synthesis and inflammation^21,22^. The COL1A1 and COL1A2 genes encode the pro-alpha 1 and pro-alpha 2 chains, respectively, to assemble the triple helix construction of collagen I. RIP1-RIP3-MLMK pathway has been identified as a critical mediator in initiating necroptosis and participates in inflammatory responses^39–41^. Evidence shows that RIP1 and RIP3 are elevated in human and experimental aneurysm tissues^23,40,42^. RIP3 deletion or RIP1 inhibition suppressed SMCS necrosis, cytokine TNF-α, IFN-β and TLR3 production, necrosis marker phospho-MLKL expression and aortic dilation^23^. Similarly, transplanting RIP3^(+/−)^ aorta into RIP3^(+/+)^ mice caused aneurysm resistance, and the underlying mechanism was RIP3 deletion, which inhibited the necrosis of SMCs and the production of inflammatory factors TNF-α and p65^25^. Necrotic vascular smooth muscle cells can release IL-1 and induce the production of IL-6, leading to vascular inflammation^43^. In aneurysm, the expression of MMPs co-localised with α-SMA is elevated^44^. α-SMA reductions facilitate the development of aneurysm pathophysiology^23^. Moreover, the MMP pathway can activate the caspase family members, leading to cell apoptosis and increase TNF-α^44^. In turn, TNF-α induces the proliferation of the HASMCs and the expression of MMP2 and MMP9^45,46^. RIP3 is involved in TNF-α-induced cell necrosis in SMCs^25^. MiR-513b-5p/COL1A1/COL1A2 signalling contributes to the progression of IA by causing HSMC necroptosis and stimulating vascular inflammation.

## Conclusion

The present results showed that miR-513b-5p/COL1A1/COL1A2, inflammation and MMPs were activated in IA. COL1A1, COL1A2, TNF-α, IL-1β and MMP9 contributed more to the ruptured IA. COL1A1 and COL1A 2 were the direct targets of miR-513b-5p. MiR-513b-5p targeted COL1A1/2 to inhibit the RIP1-RIP3-MLKL and MMP pathways, thereby enhancing the HASMC death, apoptosis, inflammation and extracellular matrix destruction.

## Supplementary Information

Supplementary Figures.

Supplementary Figures.

## Abbreviations

COL1A1: Collagen type I alpha 1 chain
COL1A2: Collagen type I alpha 2 chain
IA: Intracranial aneurysm
RA: Rupture aneurysm
UA: Unrupture aneurysm
qRT-PCR: Quantitative real-time polymerase chain Reaction
MTT: Methyl thiazolyl tetrazolium
LDH: Lactate dehydrogenase
TNF-α: Tumor necrosis factor alpha
IL-6: Interleukin 6
MMP2: Matrix metallopeptidase 2
MMP3: Matrix metallopeptidase 3
MMP9: Matrix metallopeptidase 9
TIMP4: Tissue inhibitor of metallopeptidase 4
HASMC: Human vascular smooth muscle cells
RIP3: Receptor interacting serine/threonine kinase 3
p-RIP3: Phospho receptor interacting serine/threonine kinase 3
RIP1: Receptor interacting serine/threonine kinase 1
p-RIP1: Phospho receptor interacting serine/threonine kinase 1
p-MLKL: Phospho mixed lineage kinase domain like pseudokinase
IFN-β: Interferon beta
TLR3: Toll like receptor 3
α-SMA: Alpha smooth muscle actin
GAPDH: Glyceraldehyde-3-phosphate dehydrogenase
