# Supplementary material for: MicroRNA-513b-5p targets COL1A1 and COL1A2 associated with the formation and rupture of intracranial aneurysm
Source: Sci Rep. 2021 Jul 21;11:14897. doi: 10.1038/s41598-021-94116-5 (PMC8295310; doi:10.1038/s41598-021-94116-5)
Supplement: Supplementary file 1 — Supplementary Figures. [file 41598_2021_94116_MOESM1_ESM.docx]

**supplementary information**


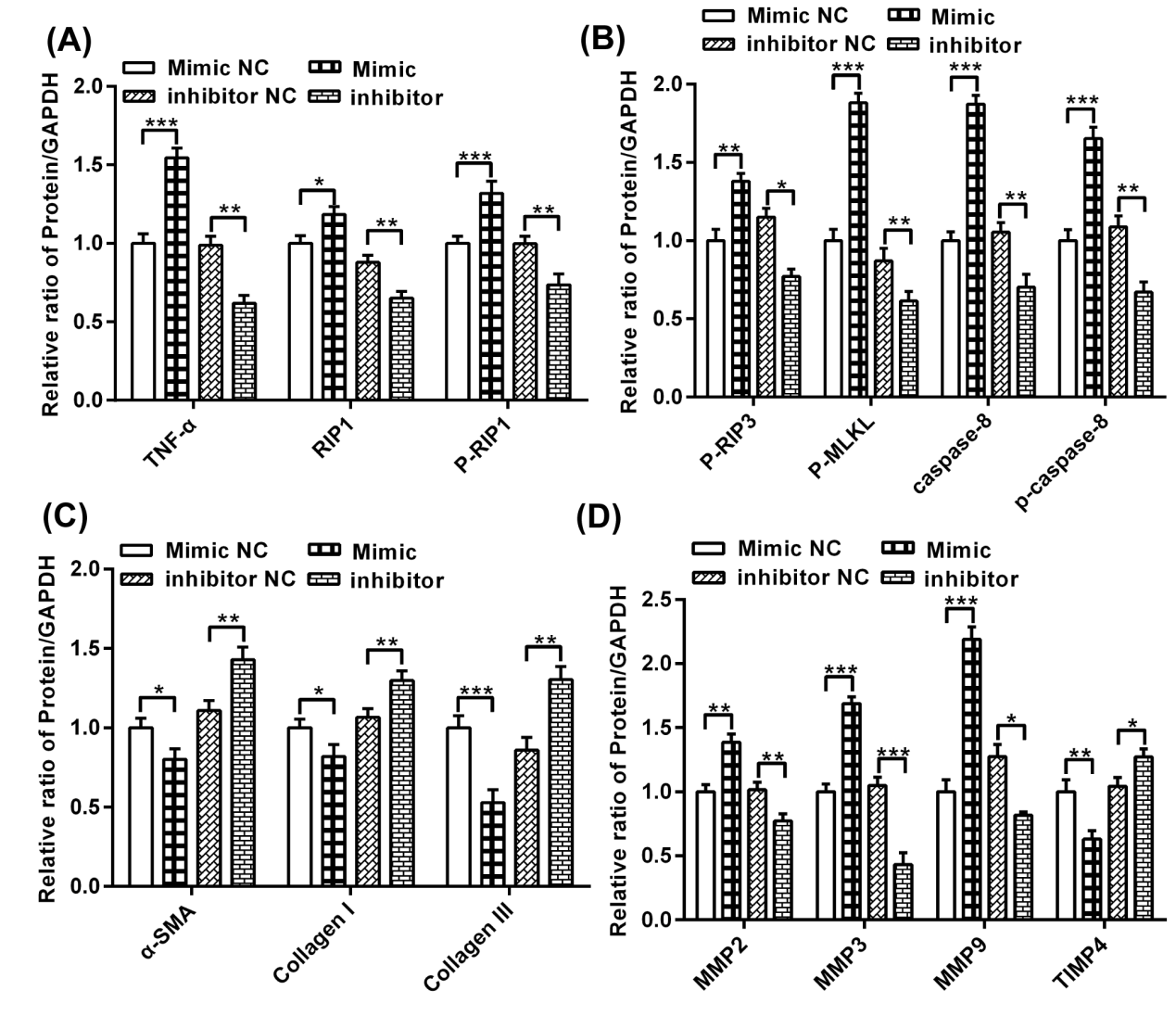


**Supplementary 1F MiR-513b-5p regulated RIP1-RIP3-MLKL and MMPs pathway.** (A) Western blot analyzed TNF-α, RIP1, phospho-RIP1 expression. (B) The expression of phospho-RIP3, phospho-MLKL, caspase-8 and phospho-caspase-8 protein. (C) The changes of α-SMA, collagen I, collagen III expression. (D) MMP2, MMP3, MMP9, and TIMP4 expression. All of the data are expressed as the mean ±standard deviation, and three independent experiments were carried out. Compared with mimic NC or inhibitor NC group, *P < 0.05, **P < 0.01, and ***P < 0.001. Student's t-test with two-sided.


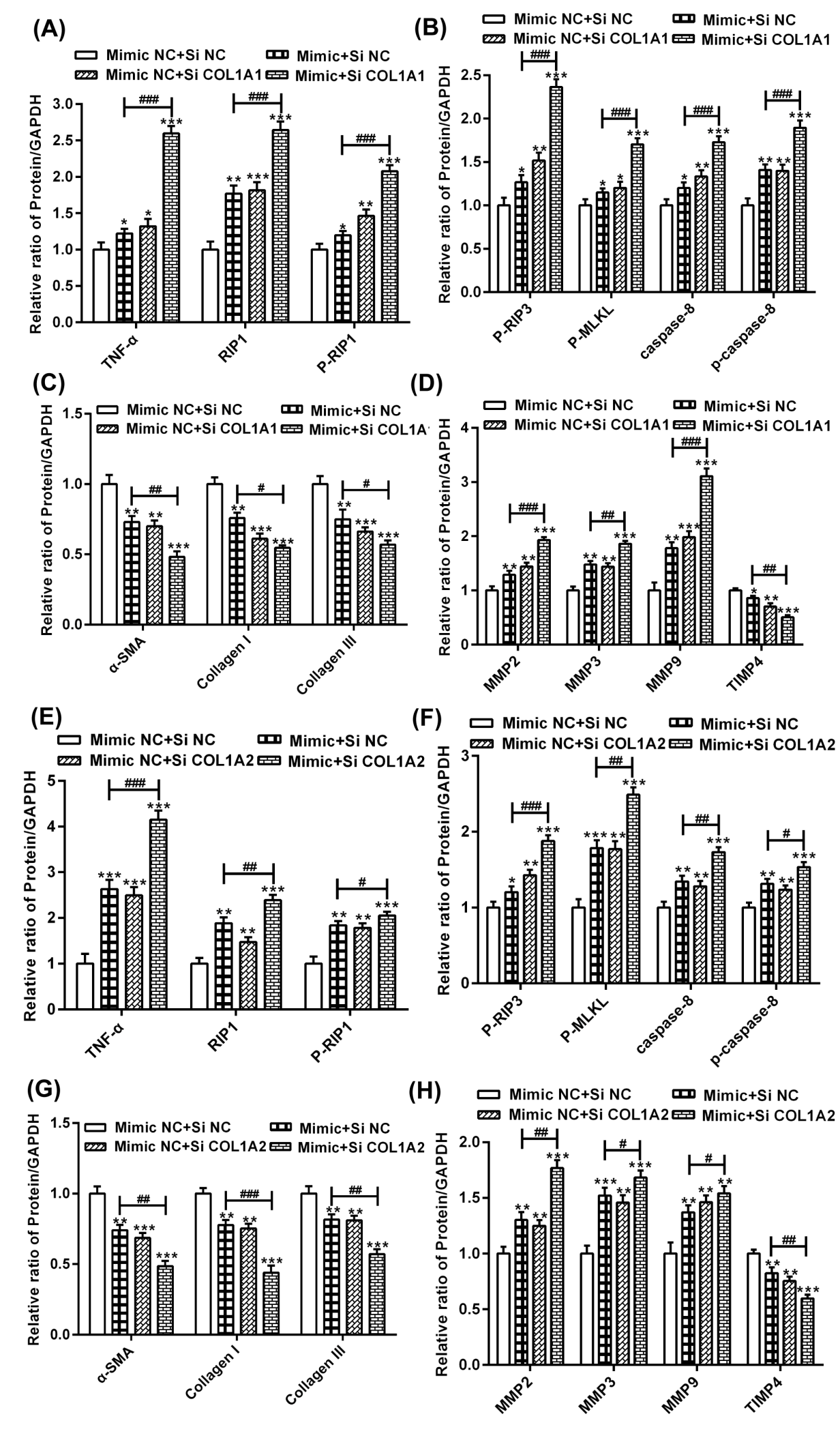


**Supplementary 2F MiR-513b-5p targets COL1A1 or COL1A2 regulated RIP1-RIP3-MLKL and MMPs pathway.** (A) MiR-513b-5p targets COL1A1 to regulate the expression of TNF-α, RIP1 and phospho-RIP1. (B) MiR-513b-5p targets COL1A1 to regulate the expression of phospho-RIP3, phospho-MLKL, caspase-8 and phospho-caspase-8 protein. (C) MiR-513b-5p targets COL1A1 to regulate the expression of α-SMA, collagen I and collagen III. (D) MiR-513b-5p targets COL1A1 to regulate the expression of MMP2, MMP3, MMP9 and TIMP4. (E) MiR-513b-5p targets COL1A2 to regulate the expression of TNF-α, RIP1 and phospho-RIP1. (F) MiR-513b-5p targets COL1A2 to regulate the expression of phospho-RIP3, phospho-MLKL, caspase-8 and phospho-caspase-8 protein. (G) MiR-513b-5p targets COL1A2 to regulate the expression of α-SMA, collagen I and collagen III. (F) MiR-513b-5p targets COL1A2 to regulate the expression of MMP2, MMP3, MMP9 and TIMP4.All of the data are expressed as the mean ±standard deviation, and three independent experiments were carried out. Compared with mimic NC+siRNA NC group, *P < 0.05, **P < 0.01, and ***P < 0.001. Compared with miR-513b-5p mimic +siRNA NC group, ^#^P < 0.05, ^##^P < 0.01, and ^###^P < 0.001.The one-way ANOVA analysis with LSD post hoc test with two-sided.
