# Supplementary material for: MicroRNA-513b-5p targets COL1A1 and COL1A2 associated with the formation and rupture of intracranial aneurysm
Source: Sci Rep. 2021 Jul 21;11:14897. doi: 10.1038/s41598-021-94116-5 (PMC8295310; doi:10.1038/s41598-021-94116-5)

Title: MicorRNA-513b-5p Targeting COL1A1 and COL1A2 Associated with Intracranial Aneurysm Formation and Rupture: a Longitudinal Study

Runing Title: Role of miRNA-513b-5p targeting COL1A1 and COL1A2 in IA

Authors: Zheng Zheng[1,2†], Yan Chen[1,3†], Yinzhou Wang[1,2], Yongkun Li[1,2], Qiong Cheng[1,2] *

Affiliations

[1]Shengli Clinical Medical College, Fujian Medical University, Fuzhou, 350001, P.R. China,

[2]The Department of Neurology, Fujian Provincial Hospital, Fuzhou, 350001, P.R. China

[3]The Department of Geriatric Medicine, Fujian Provincial Hospital, Fuzhou, 350001, P.R. China.

[†]**Co-first Author**: Zheng Zheng, Yan Chen.

***Corresponding Author:** Qiong Cheng, Shengli Clinical Medical College, Fujian Medical University, Fuzhou, 350001, P.R. China; the Department of Neurology, Fujian Provincial Hospital, NO.134, Dongjie road, Fuzhou, 350001, P.R. China; Tel: 86-13509357396, Email: chengqiong13@126.com.

Authors' E-mail:

Zheng Zheng, fjslzz@fjmu.edu.cn; Yan Chen, cy514zz@163.com;

Yinzhou Wang, wphd@163.com; Yongkun Li, 13599081830@139.com;

Qiong Cheng, chengqiong13@126.com;

Figure 3D the original western blots (Some blots were Rotation Angle)

GAPDH

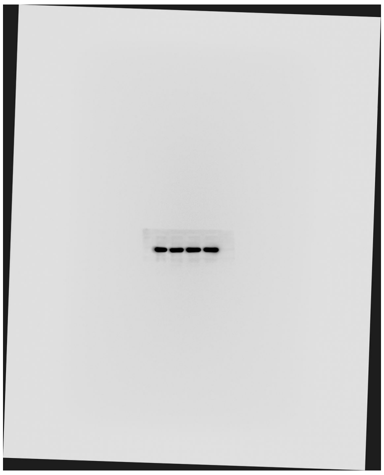

TNF-α

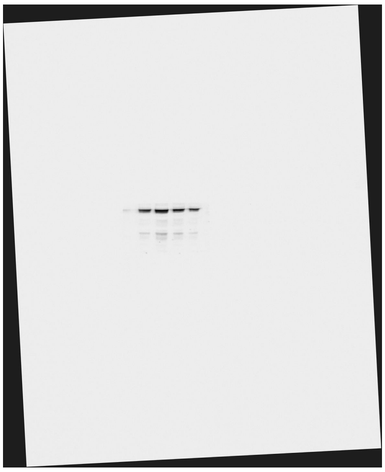

RIP1

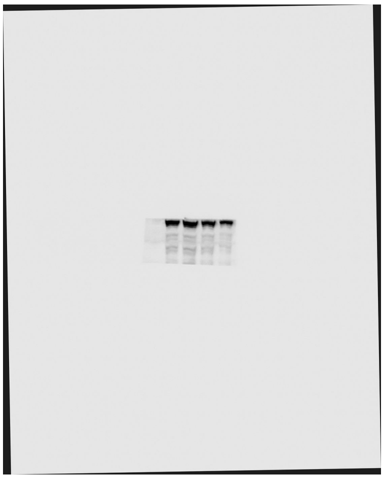

P-RIP1

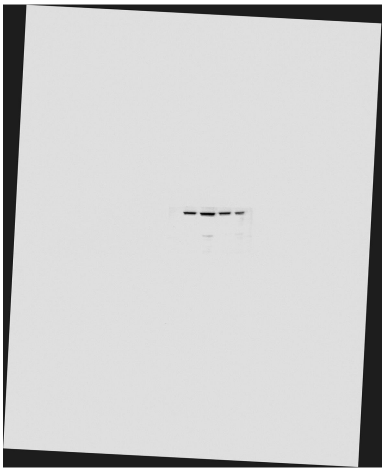

P-RIP3

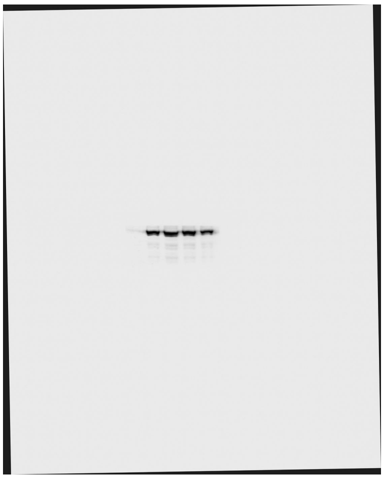

P-MLKL

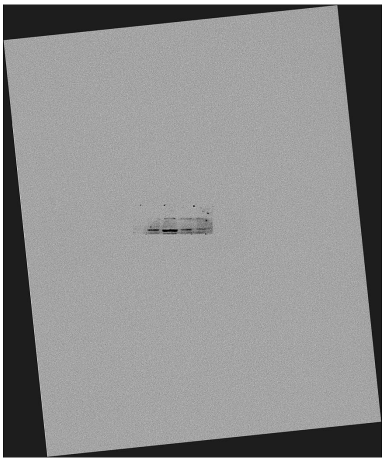

caspase-8

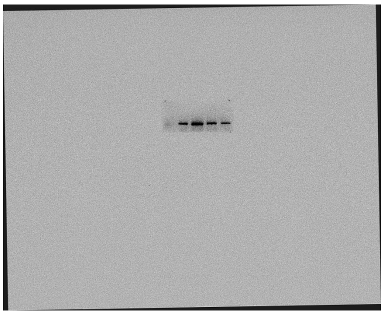

p-caspase-8

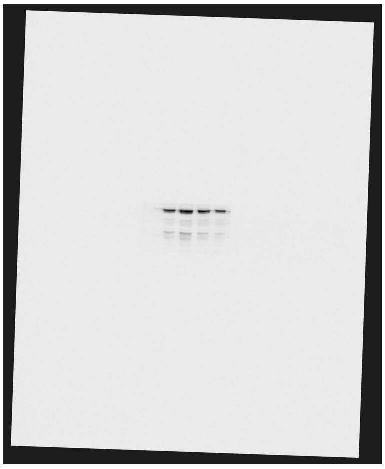

Figure 3E the original western blots

GAPDH

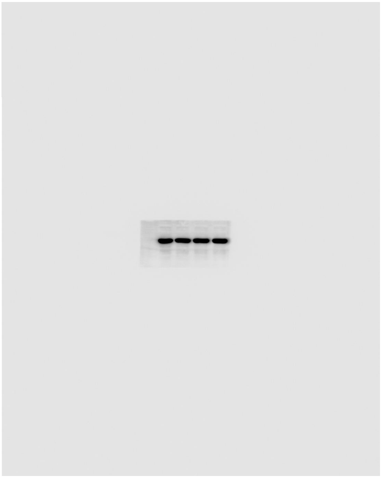

α-SMA

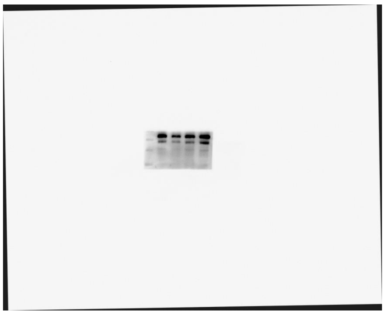

collagen I

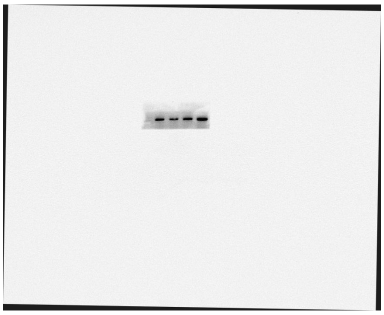

MMP2

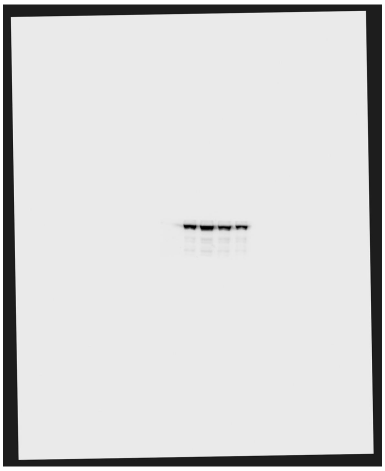

MMP3

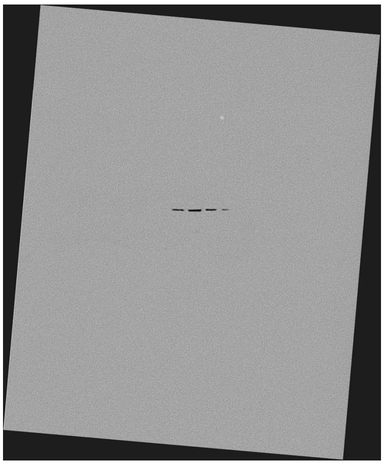

MMP9

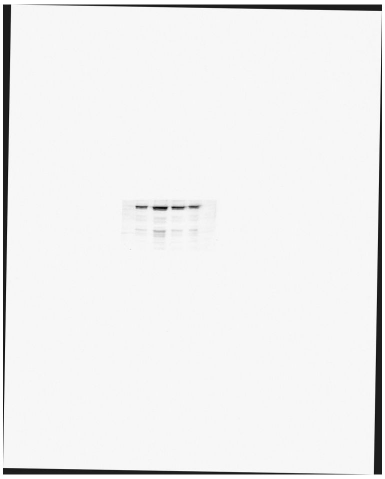

TIMP4

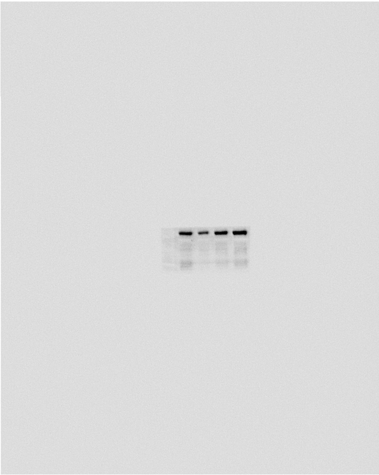

collagen III

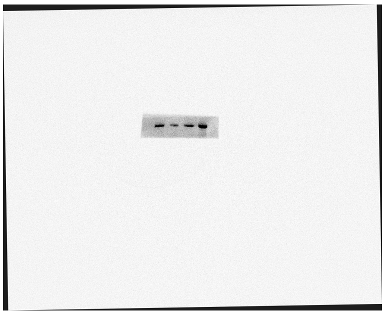

Figure 4A the original western blots

GAPDH

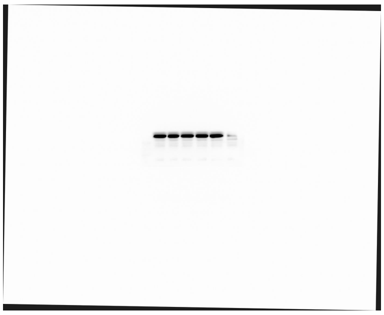

COL1A1

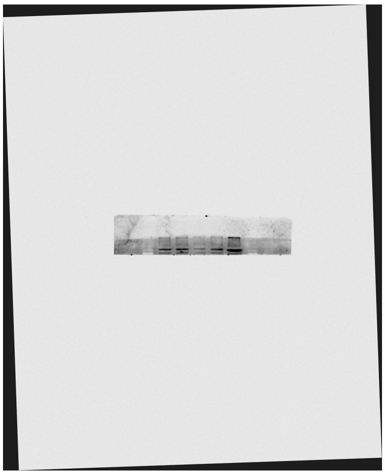

Figure 4H the original western blots

GAPDH

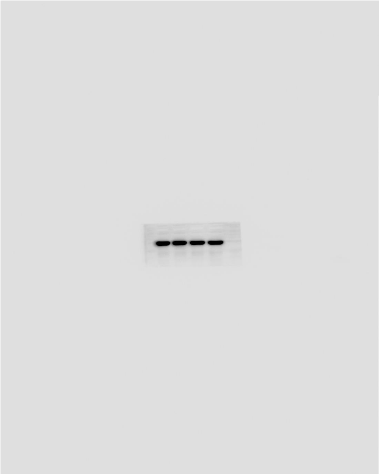

TNF-α

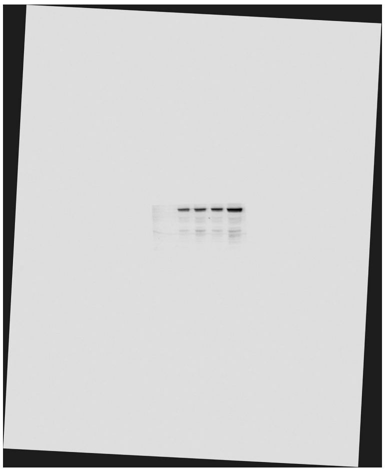

RIP1

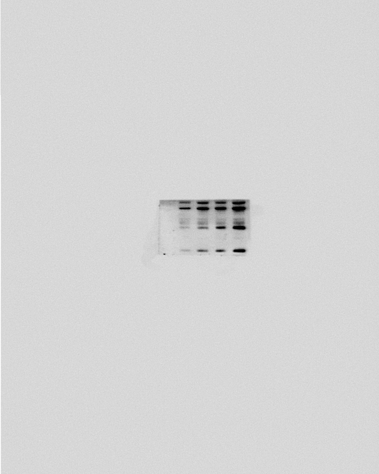

P-RIP1

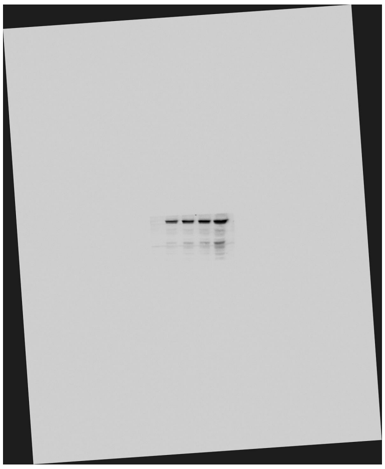

P-RIP3

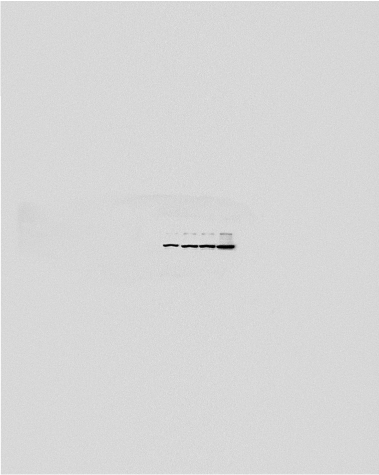

P-MLKL

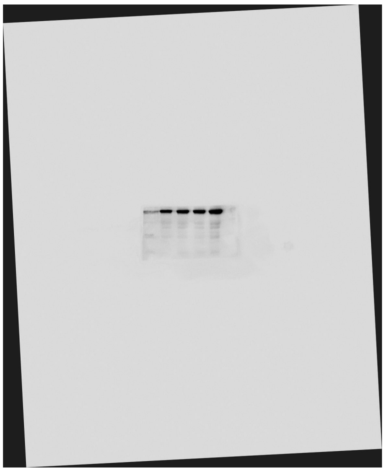

caspase-8

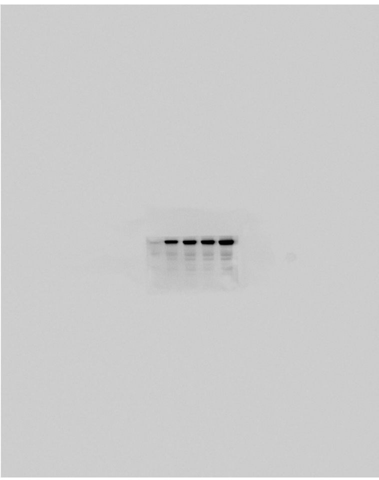

p-caspase-8

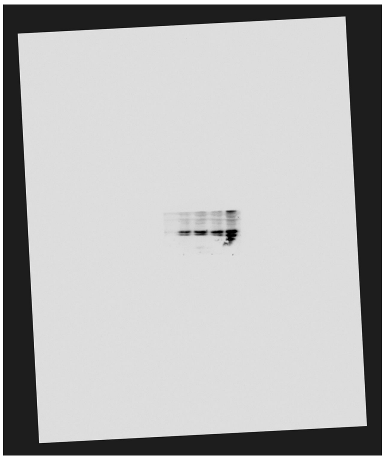

Figure 4I the original western blots

GAPDH

α-SMA

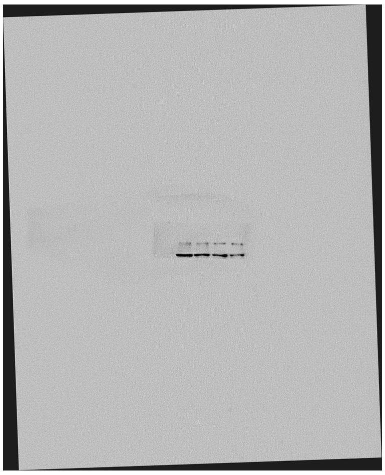

collagen I

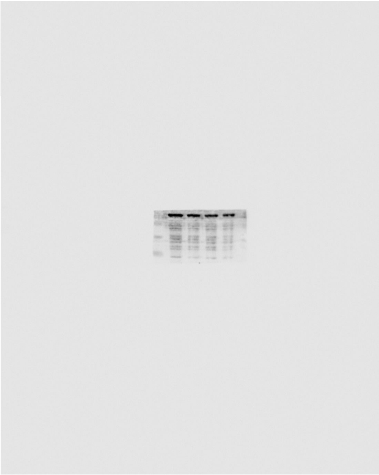

collagen III

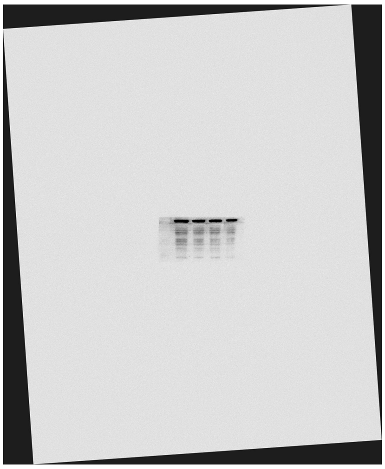

MMP2

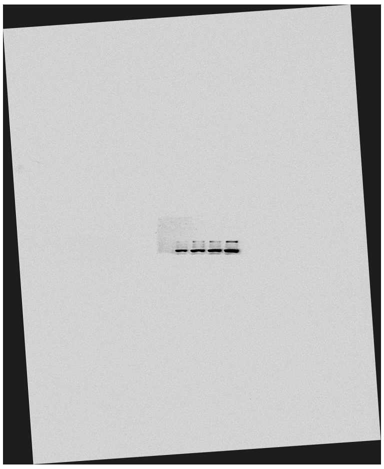

MMP3

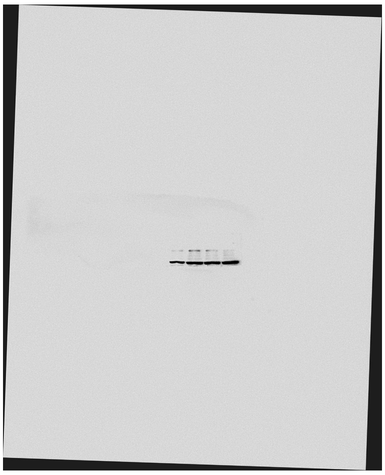

MMP9

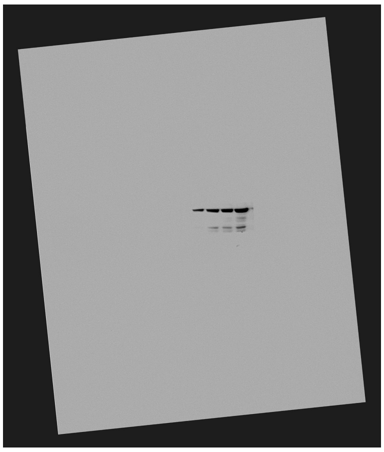

TIMP4

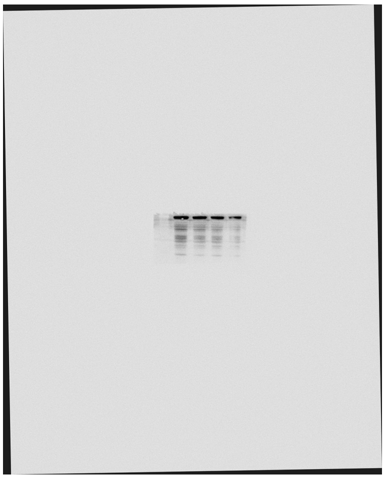

Figure 5A the original western blots

GAPDH

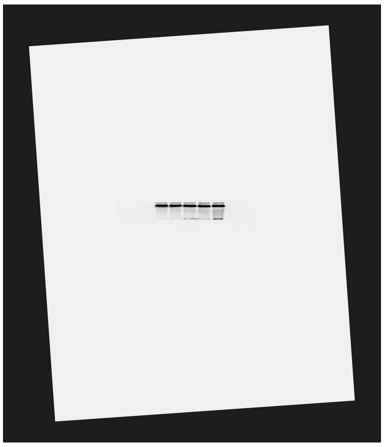

COL1A2

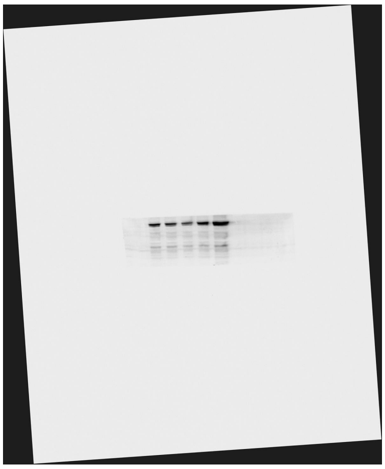

Figure 5H the original western blots

GAPDH

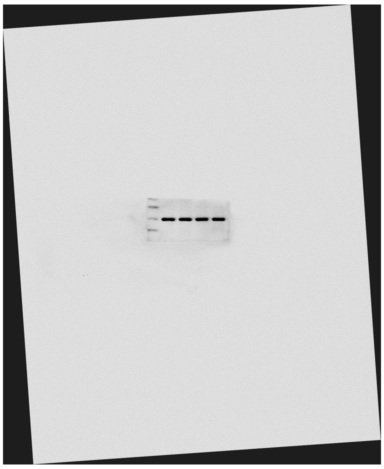

TNF-α

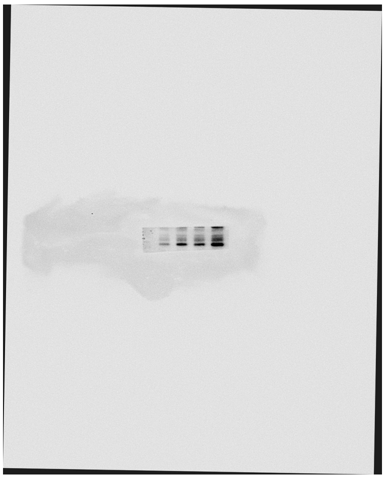

RIP1

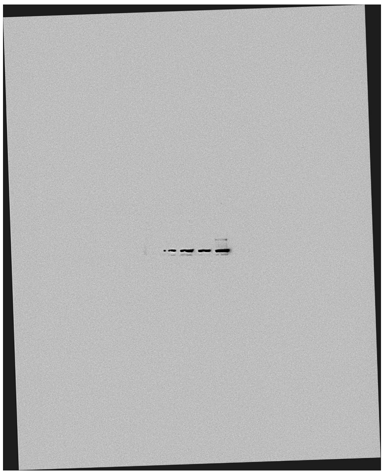

P-RIP1

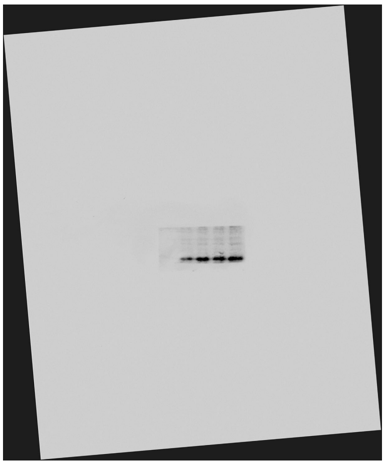

P-RIP3

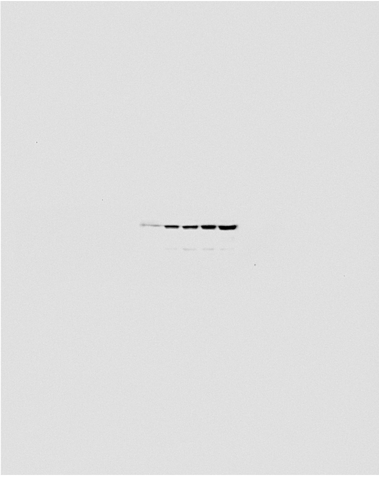

P-MLKL

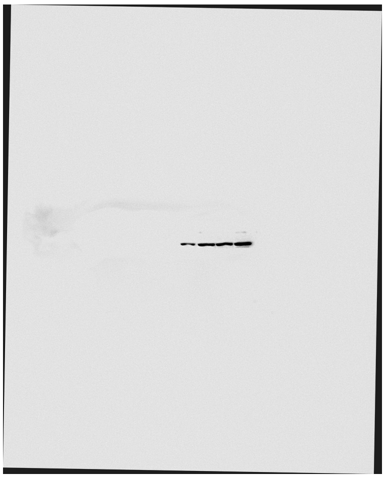

caspase-8

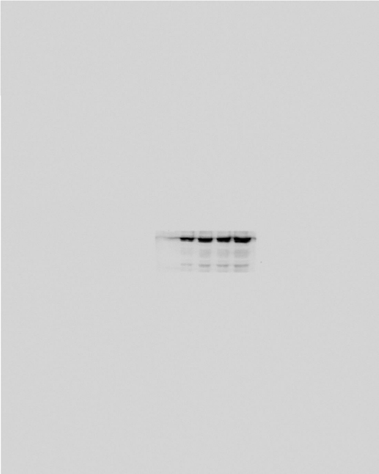

p-caspase-8

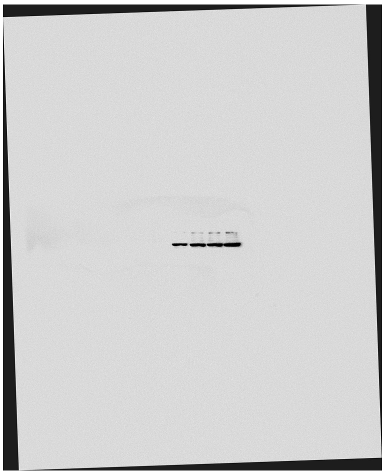

Figure 5I the original western blots

GAPDH

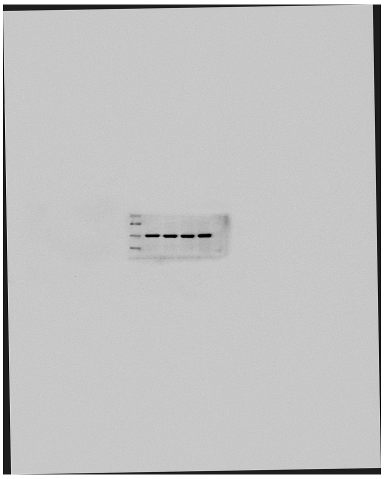

α-SMA

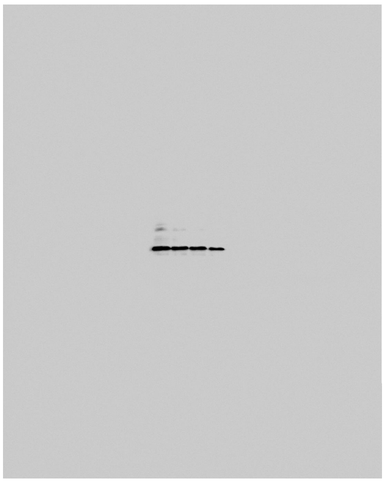

collagen I

collagen III

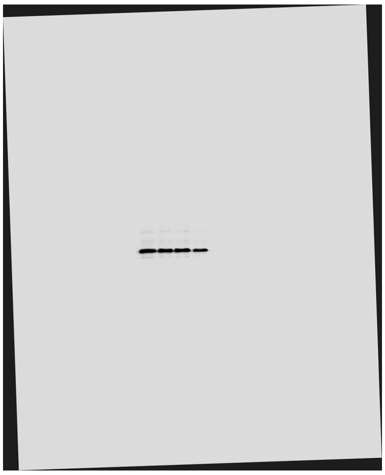

MMP2

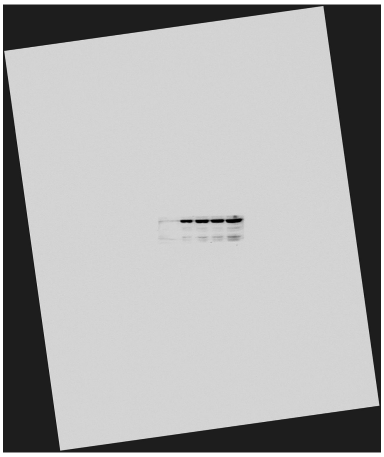

MMP3

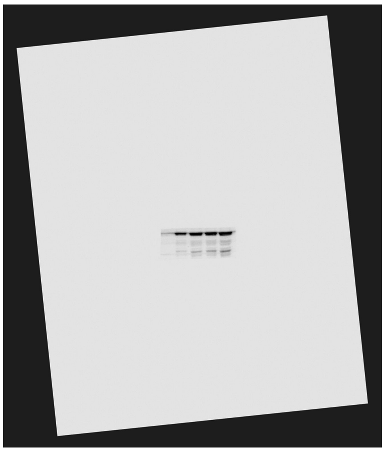

MMP9

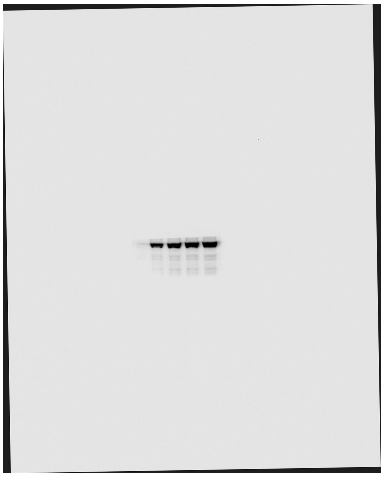

TIMP4

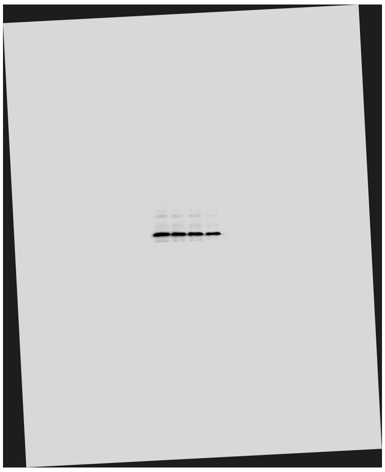

Figure 6D the original western blots

GAPDH

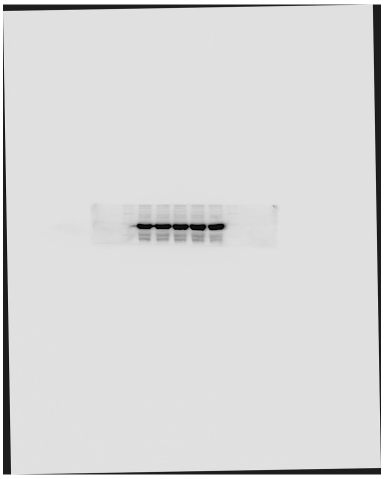

RIP1

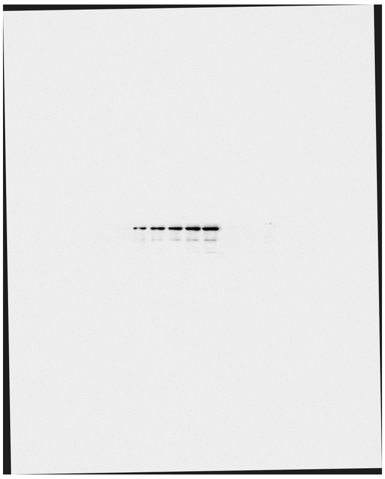

P-RIP1

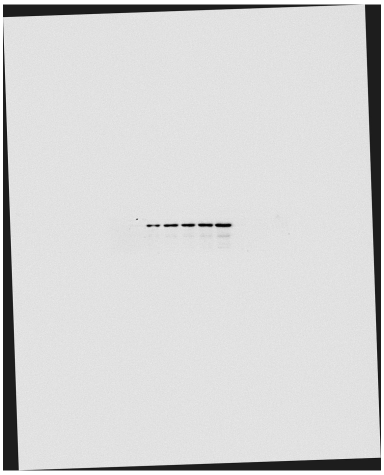

P-RIP3

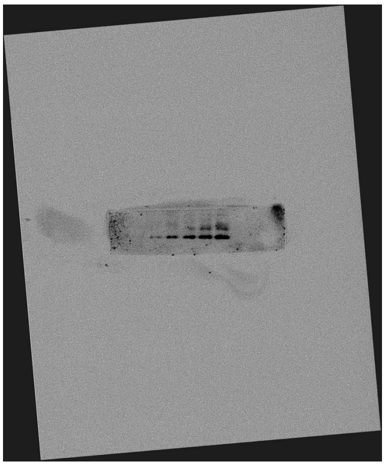

P-MLKL

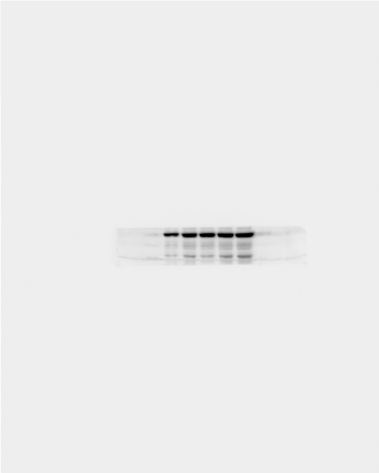

Figure 6E the original western blots

GAPDH

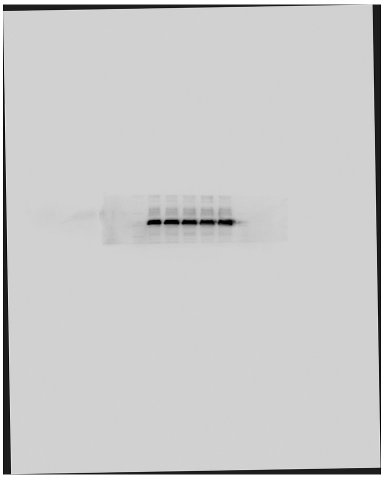

collagen I

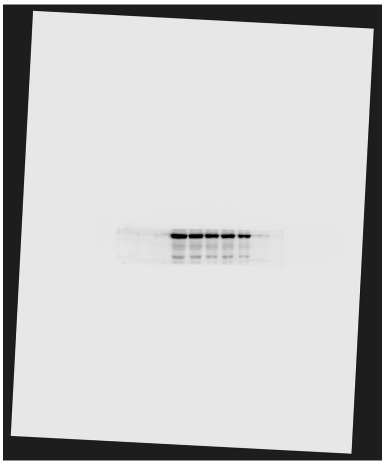

MMP2

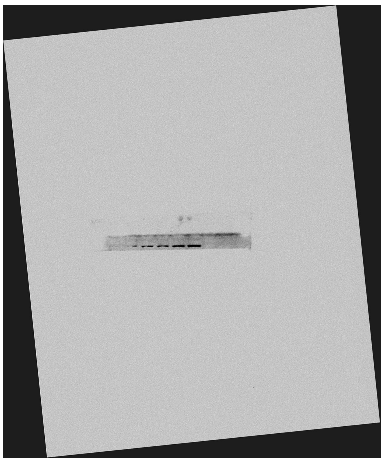

MMP9

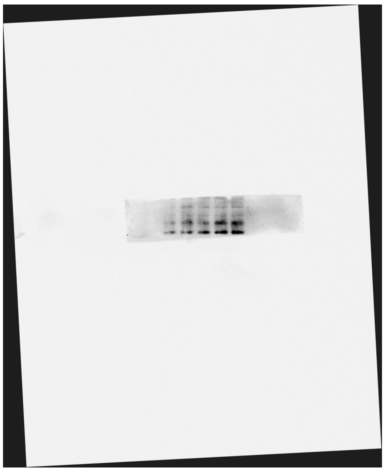

TIMP4

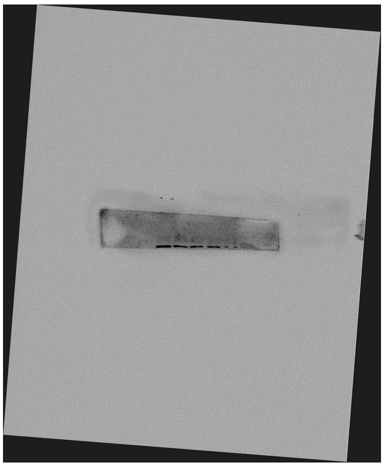

Figure 7D the original western blots

GAPDH

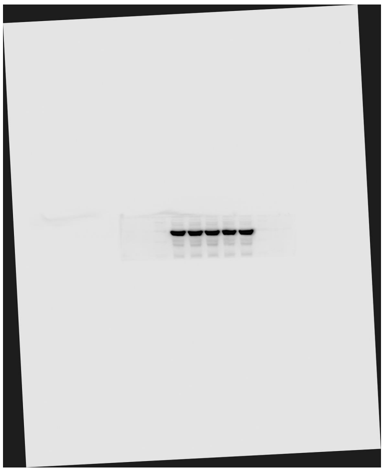

RIP1

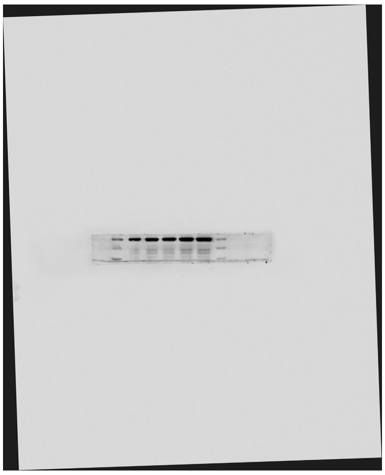

P-RIP1

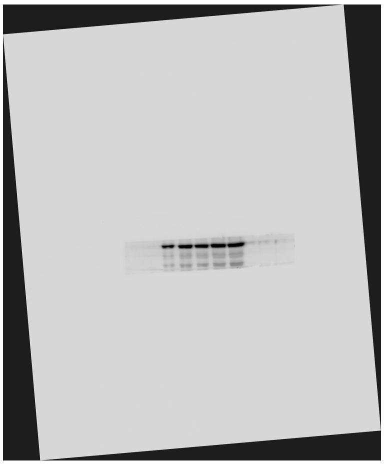

P-RIP3

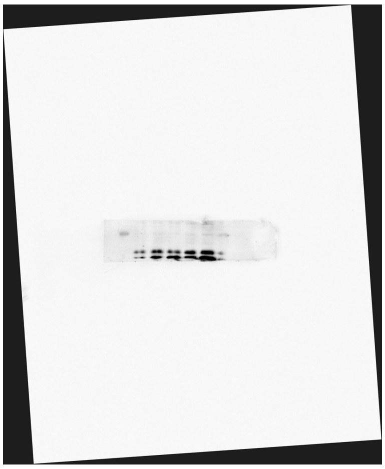

P-MLKL

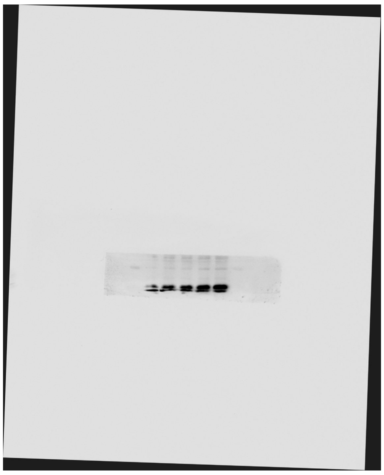

Figure 7E the original western blots

GAPDH

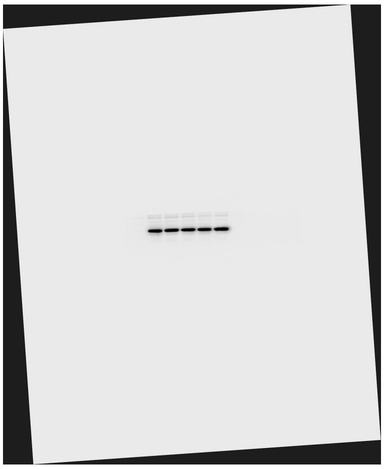

collagen I

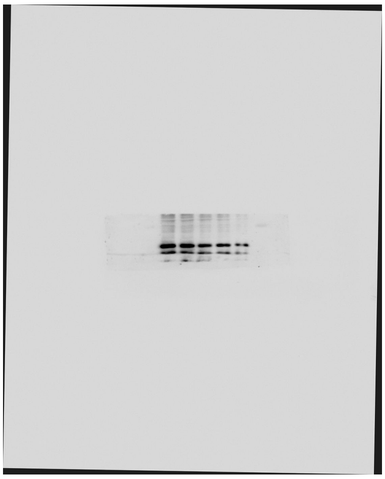

MMP2

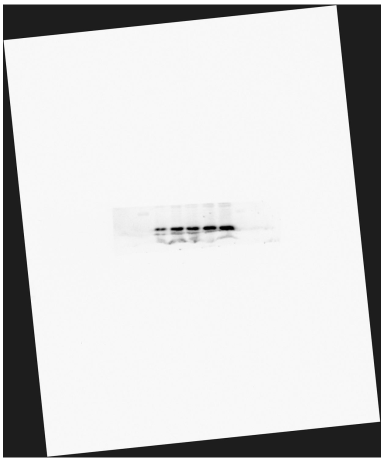

MMP9

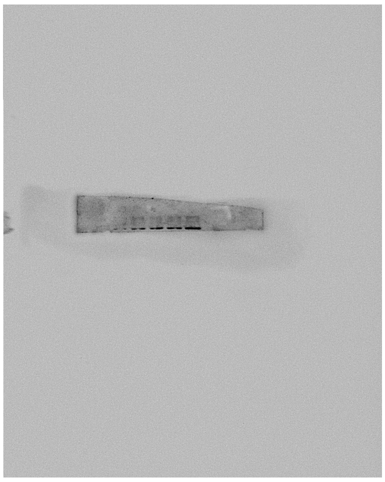

TIMP4

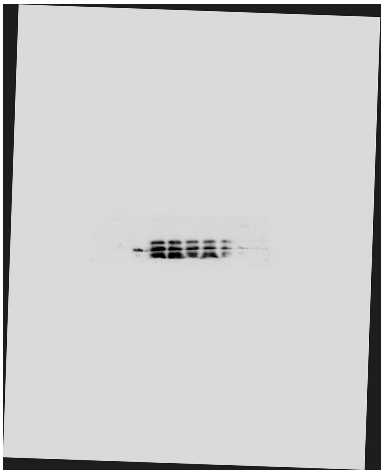

Supplement: Supplementary file 2 — Supplementary Figures. [file 41598_2021_94116_MOESM2_ESM.pdf]
